# Unveiling the interplay between NSAID-induced dysbiosis and autoimmune liver disease in children: insights into the hidden gateway to autism spectrum disorders. Evidence from *ex vivo*, *in vivo*, and clinical studies

**DOI:** 10.3389/fncel.2023.1268126

**Published:** 2023-10-31

**Authors:** Doaa I. Mohamed, Hebatallah H. Abo Nahas, Asmaa M. Elshaer, Dalia Alaa El-Din Aly El-Waseef, Omnyah A. El-Kharashi, Soha M. Y. Mohamed, Yasmine Gamal Sabry, Riyad A. Almaimani, Hussain A. Almasmoum, Abdulmalik S. Altamimi, Ibrahim Abdel Aziz Ibrahim, Samar Z. Alshawwa, Mariusz Jaremko, Abdul-Hamid Emwas, Essa M. Saied

**Affiliations:** ^1^Department of Clinical Pharmacology and Therapeutics, Faculty of Medicine, Ain Shams University, Cairo, Egypt; ^2^Physiology Department, Faculty of Science, Suez Canal University, Ismailia, Egypt; ^3^Department of Histology and Cell Biology, Faculty of Medicine, Ain Shams University, Cairo, Egypt; ^4^Physiology Department, Faculty of Medicine, Ain Shams University, Cairo, Egypt; ^5^Department of Biochemistry, Faculty of Medicine, Umm Al-Qura University, Makkah, Saudi Arabia; ^6^Department of Laboratory Medicine, Faculty of Applied Medical Sciences, Umm Al-Qura University, Makkah, Saudi Arabia; ^7^Department of Pharmaceutical Chemistry, College of Pharmacy, Prince Sattam Bin Abdulaziz University, Alkharj, Saudi Arabia; ^8^Department of Pharmacology and Toxicology, Faculty of Medicine, Umm Al-Qura University, Makkah, Saudi Arabia; ^9^Department of Pharmaceutical Sciences, College of Pharmacy, Princess Nourah bint Abdulrahman University, Riyadh, Saudi Arabia; ^10^Smart-Health Initiative and Red Sea Research Center, Division of Biological and Environmental Sciences and Engineering, King Abdullah University of Science and Technology, Thuwal, Saudi Arabia; ^11^Advanced Nanofabrication Imaging and Characterization Center, King Abdullah University of Science and Technology, Core Labs, Thuwal, Saudi Arabia; ^12^Chemistry Department, Faculty of Science, Suez Canal University, Ismailia, Egypt; ^13^Institute for Chemistry, Humboldt Universität zu Berlin, Berlin, Germany

**Keywords:** autism spectrum disorders, NSAIDs, gut microbiota, dysbiosis, autoimmune hepatitis, inflammasomes, prognostic biomarkers, clinical study

## Abstract

Autism spectrum disorders (ASD) represent a diverse group of neuropsychiatric conditions, and recent evidence has suggested a connection between ASD and microbial dysbiosis. Immune and gastrointestinal dysfunction are associated with dysbiosis, and there are indications that modulating the microbiota could improve ASD-related behaviors. Additionally, recent findings highlighted the significant impact of microbiota on the development of autoimmune liver diseases, and the occurrence of autoimmune liver disease in children with ASD is noteworthy. In the present study, we conducted both an *in vivo* study and a clinical study to explore the relationship between indomethacin-induced dysbiosis, autoimmune hepatitis (AIH), and the development of ASD. Our results revealed that indomethacin administration induced intestinal dysbiosis and bacterial translocation, confirmed by microbiological analysis showing positive bacterial translocation in blood cultures. Furthermore, indomethacin administration led to disturbed intestinal permeability, evidenced by the activation of the NLRP3 inflammasomes pathway and elevation of downstream biomarkers (TLR4, IL18, caspase 1). The histological analysis supported these findings, showing widened intestinal tight junctions, decreased mucosal thickness, inflammatory cell infiltrates, and collagen deposition. Additionally, the disturbance of intestinal permeability was associated with immune activation in liver tissue and the development of AIH, as indicated by altered liver function, elevated ASMA and ANA in serum, and histological markers of autoimmune hepatitis. These results indicate that NSAID-induced intestinal dysbiosis and AIH are robust triggers for ASD existence. These findings were further confirmed by conducting a clinical study that involved children with ASD, autoimmune hepatitis (AIH), and a history of NSAID intake. Children exposed to NSAIDs in early life and complicated by dysbiosis and AIH exhibited elevated serum levels of NLRP3, IL18, liver enzymes, ASMA, ANA, JAK1, and IL6. Further, the correlation analysis demonstrated a positive relationship between the measured parameters and the severity of ASD. Our findings suggest a potential link between NSAIDs, dysbiosis-induced AIH, and the development of ASD. The identified markers hold promise as indicators for early diagnosis and prognosis of ASD. This research highlights the importance of maintaining healthy gut microbiota and supports the necessity for further investigation into the role of dysbiosis and AIH in the etiology of ASD.

## 1. Introduction

Autism spectrum disorders (ASD) are a complex array of neuropsychiatric conditions that can affect individuals in unique ways. These conditions are distinguished by difficulties in communication, social interactions and stereo-typed behavioral patterns. While the prevalence of ASD has increased significantly since its initial recognition, the current rate of 1 in 59 individuals affected by this condition is truly staggering, with a male predominance (Baio et al., 2018). While the exact cause of ASD remains a mystery, researchers have identified genetic and environmental factors as potential contributors. In fact, it is estimated that up to 20% of ASD cases can be attributed to genetic factors alone (Abrahams and Geschwind, 2008). Immune dysfunction and gastrointestinal inflammation are common occurrences in individuals with ASD, contributing to the intricate array of symptoms associated with the disorder (Belkaid and Hand, 2014). The gut-brain axis represents a complex and dynamic physiological network through which gut microbiota interacts with the (CNS) via intricate neuronal, endocrine, and immunological signaling pathways. The communication between these two systems is bidirectional, and thus, the influence of the brain on the gut microbial content. The microbiota’s role in autoimmune liver diseases has gained recognition in recent studies. Similarly, the influence of the intestinal microbiota on brain function also warrants further investigation. This intricate interplay between the gut and the brain involves multiple molecular mechanisms, including the production of various neuroactive substances such as neurotransmitters, neuropeptides, and hormones by both the gut and the CNS. These molecules play critical roles in modulating various physiological and behavioral responses, including appetite regulation, stress response, immune function, and mood regulation. Overall, a comprehensive understanding of the gut-brain axis is essential for elucidating the pathophysiology of various neurological and gastrointestinal disorders and developing effective therapeutic interventions (Collins and Bercik, 2009; Abo Nahas et al., 2022; Helmy et al., 2023).

The synthesis of short-chain fatty acids by the microbiota has been found to negatively impact the integrity of both the intestinal and blood–brain barriers (Logsdon et al., 2018). Experimental studies utilizing a dysbiosis mouse model have demonstrated that com-promised intestinal barrier function can result in the translocation of bacterial metabolites and immunological mediators from the gastrointestinal tract into the bloodstream. This may subsequently activate microglia and cause neuroinflammation (Bruce-Keller et al., 2015). Accumulating animal studies have shown that dysbiosis or alterations in the gut microbiota composition can influence neurodevelopment and behavior. In recent years, there has been growing evidence of microbial dysbiosis in individuals with ASD. Dysbiosis in ASD is associated with immune and gastrointestinal dysfunction. Notably, there is emerging evidence that the microbiota modifications in ASD can lead to improvements in behaviors (Kang et al., 2017).

Nonsteroidal anti-inflammatory drugs (NSAIDs) are widely prescribed medications used globally to alleviate pain, inflammation, and fever. These drugs exert their pharmacological effects by inhibiting the enzyme cyclooxygenase (COX), which plays a crucial role in the biosynthesis of various proteinoids from arachidonic acid (AA). Proteinoids include prostaglandin E2, PGF2a, PGD2, prostacyclin (PGI2), and thromboxane (TxA2). COX consists of two isozymes, COX-1 and COX-2, which have distinct physiological activities, gene regulation, and regulatory sequences (Maseda and Ricciotti, 2020; El Azab et al., 2021).

Infants born preterm and with low birth weight have an increased risk of developing symptomatic patent ductus arteriosus (PDA), a vascular link between the lungs and heart that typically closes shortly after birth. However, in premature newborns, the PDA remains open and can lead to serious and potentially life-threatening consequences (Evans et al., 2021). Addressing cyclooxygenase inhibitors, numerous techniques have been pursued for PDA treatment, including prophylactic therapy within the first 24 h of birth for at risk neonates (Fowlie et al., 2010). The prophylactic administration of indomethacin, a non-selective cyclooxygenase inhibitor, has been shown to provide short-term clinical benefits. However, the efficacy of indomethacin (NSAIDs) in preterm infants with symptomatic PDA has not been thoroughly investigated (Ohlsson et al., 2020). The liver is viewed as a primary target for gut microbes because of the anatomical and functional linkages between the gut and liver, this distinctive anatomical and physio-logical relationships such as communication through the biliary tract, systemic circulation, and portal vein, strongly imply a close connection between the intestinal micro-biome and the liver (Elshaer et al., 2019). Recent investigations have illuminated the crucial role of the microbiota in the development of autoimmune liver conditions, which encompass autoimmune hepatitis (AIH), primary biliary cholangitis, and primary sclerosing cholangitis (Bogdanos et al., 2014). A correlation has been established between the microbiome and autoimmune disorders, specifically systemic lupus erythematosus (Katz-Agranov and Zandman-Goddard, 2017), inflammatory bowel disease (Franzosa et al., 2019), and rheumatoid arthritis (LaRusso et al., 2017; Bergot et al., 2019). The importance of microbial metabolites in modulating the impact of the microbiota on host immune responses and facilitating host-microbiota interactions is gaining more recognition. These metabolites are now understood to be pivotal factors in these processes. The repertoire of gut microbial metabolites encompasses an extensive assortment of molecules, which includes, but is not limited to, short-chain fatty acids, vitamins, secondary bile acids, and neurotransmitters (Kummen and Hov, 2019). The microbiome plays a pivotal role in the modulation of inflammatory and immune responses through its capacity to serve as a catalyst for triggering factors (Kummen and Hov, 2019).

The Janus kinase/signal transducers and activators of transcription (JAK/STAT) pathway functions as an intermediary mechanism that holds significant importance in the control of inflammatory progressions. It accomplishes this by facilitating the transmission of signals among surface receptors and cytokines, including interferons (IFNs) and interleukins (ILs) (Schindler et al., 2007). Upon ligand binding, the cognate receptors become activated, leading to the activation of JAKs. JAKs then undergo mutual phosphorylation and phosphorylate the associated receptors. This interaction triggers the activation of STAT proteins, which can function as transcription factors or activate downstream signaling pathways. JAK1, a tyrosine kinase with broad distribution, assumes a pivotal function in the transmission of signals mediated by cytokines, including IFNα/β, IFNγ, IL-2, IL-6, and IL-10 (O’Shea et al., 2015). Heterozygous mutations in the JAK1 gene have been shown to cause JAK–STAT hyperactivity, leading to an autosomal dominant disorder characterized by multiorgan immune dysregulation. The JAK–STAT signaling pathway is widely acknowledged as a pivotal regulator of diverse cellular processes, including immune responses, cell growth, differentiation, and survival. The occurrence of disturbances in this particular pathway has been linked to the emergence and progression of various diseases, encompassing immune-mediated conditions and malignancies. An increasing body of empirical evidence suggests an increasing amount of evidence pointing towards the involvement of neu-ro-inflammation in both autism and 22q11.2 deletion syndrome. Patients presenting with concurrent manifestations of both conditions exhibit heightened levels of pro-inflammatory cytokines, with a notable emphasis on interleukin-6 (IL-6) which seems to exert a significant influence (Jerez et al., 2012). Thus, the identification of aberrant JAK–STAT signaling and elevated proinflammatory cytokine levels in individuals with neurodevelopmental disorders highlights the potential role of immune dysregulation and neuroinflammation in these conditions. Understanding the molecular mechanisms underlying these processes may facilitate the development of novel therapeutic strategies for the treatment of these disorders (Jerez et al., 2012).

In a study conducted by Takeichi et al. (2022), the H596D mutation in JAK1 was identified as a critical factor contributing to chronic systemic inflammation. The aforementioned conclusion was derived from clinical findings and corroborated by empirical evidence obtained from a genetically engineered mouse model. The patient with this mutation displayed an inflammatory skin phenotype consistent with autoinflammatory keratinization disease, accompanied by extracutaneous manifestations such as hepatitis and autism. Similarly, the mice with the Jak1 mutation exhibited signs of inflammation affecting both the skin and the liver. Notably, significantly increased levels of mRNA for IL-6 and other molecules associated with the IL-6 inflammatory pathway were observed in liver biopsy specimens obtained from these mice. Moreover, the results of *in vitro* experiments conducted on mouse cerebellar granule cells demonstrated that heightened expression of IL-6 disrupted the process of cellular adhesion and migration. This disruption subsequently resulted in an imbalance between excitatory and inhibitory circuits. Adding to the significance of JAK1, researchers have observed an upregulation of JAK–STAT signaling in children with autism, further emphasizing its importance (O’Shea et al., 2015; Ahmad et al., 2017). Growing proof indicates that immune dysfunction may have a significant influence on the development of neurons and the subsequent outcomes of autism (Lintas and Persico, 2009). Numerous studies have provided evidence highlighting the significance of pro-inflammatory cytokines, namely IL1, IL-6, IL-18, and TNFα, in the development of atypical behavior in children diagnosed with autism spectrum disorder. Additionally, the involvement of antinuclear antibodies (ANA), anti-smooth muscle antibodies (ASMA), and antimitochondrial antibodies as peripheral inflammatory mediators has been identified as triggers in autoimmune liver diseases (Ghonaim et al., 2005). The NLRP3 inflammasome, which serves as a platform for caspase-1 activation, is significantly involved in the regulation of hepatic inflammation and fibrosis (Wree et al., 2018).

Based on the aforementioned facts, the present study aimed to unravel the possible association between inflammatory markers, autoimmune liver disease, and autism, with a particular focus on exploring the possible role of dysbiosis in mediating this axis.

## 2. Materials and methods

### 2.1. Drugs and reagents

Indomethacin (Sigma-Chemical Co., Cairo, Egypt) was supplied as a white yellow odorless powder. The drug was suspended in a small amount of tween 80 and distilled water for oral use. Diet, Rat chow: (20% proteins, 10% fat, 70% carbohydrates) in the form of Pellets was obtained from Meladco (Animal Food, Egypt).

### 2.2. Animals and grouping

Forty male Wistar rats, weighing between (130–200) for juvenile and (250-400 g) for adult rats, were obtained from the Holding company for biological products & Vaccines VACCERA in Helwan, Egypt. This study was conducted at the department of pharmacology and Medical Research Center, Faculty of Medicine, Ain Shams University, Egypt. The rats were given a minimum of one week to acclimate to the laboratory conditions. They were housed in cages with a 12-h light/dark cycle, with lights on at 6 AM, and the temperature was maintained at 25°C. The rats were housed in groups of three animals per cage for a duration of four weeks. To ensure equal distribution, the rats were divided into two main groups based on their age;

- Juvenile rats (seven weeks old) which were subdivided into:
  1. Control group: received only distilled water with tween 80.
  2. Indomethacin-treated group: Received Indomethacin (3 mg/kg/day) dissolved in tween 80 given for 4 weeks by oral gavage.
- Adult rats (nine months old) which were subdivided into:
  1. Control group: received only distilled water with tween 80.
  2. Indomethacin-treated group: Received Indomethacin (3 mg/kg/day) dissolved in tween 80 given for 4 weeks by oral gavage.

All animals underwent intraperitoneal anesthesia using thiopental sodium at a dose of 100 mg/kg. Thiopental sodium is a barbiturate drug commonly used for induction and maintenance of anesthesia in laboratory animals. The choice of thiopental sodium as the anesthetic agent is likely due to its rapid onset of action and relatively short duration of effect. It allows for smooth and controlled anesthesia induction (Mohamed et al., 2020).

### 2.3. Induction of intestinal dysbiosis

Rats were allowed to feed normally every 5 days during the study. Intestinal dysbiosis was induced as previously reported by oral administration of indomethacin dissolved in tween 80 (3 mg/kg/day) given for 4 weeks by oral gavage for adult and juvenile rats (Pearce et al., 2018).

### 2.4. Blood culture

For blood culture analysis, two blood samples of 1 mL each were collected from each rat. The first sample was obtained from the ophthalmic venous plexus using the retro-orbital approach prior to the commencement of the experiment. The second sample was collected from the heart using a sterile disposable needle and syringe. Collection of blood was performed under complete aseptic conditions. Sterile gloves were worn. Collected blood was inoculated into neonatal 8 mL HiSafe Blood Culture System (Himedia, India). To initiate the blood culture process, the top of the screw cap was removed, and the blood sample was promptly transferred into culture bottles by puncturing the rubber stopper with a needle and injecting the blood. Gentle inversion of the bottle (2–3 times) ensured proper mixing of the contents. For aerobic conditions, one of the culture bottles was ventilated using a sterile venting needle equipped with a membrane filter. In con-trast, no ventilation was performed for the bottles intended for anaerobic cultures. The upright position of the bottles was maintained during their incubation at 37°C for 24 h, followed by an additional seven days. Additionally, 2 mL of the gathered blood samples were introduced into lavender-top EDTA-treated CBC tubes and subsequently preserved at −80°C for future biochemical analyzes (Kumar et al., 2017).

### 2.5. Isolation of pathogens

#### 2.5.1. Microbial growth

Microbial growth was usually evident within 48 h, denoted by the turbidity of fluid in the bottles or growth on the solid phase. Further incubation for 7 days was required to confirm negative results. Identification of the microorganisms present in the culture required sub-culturing on appropriate media. The isolated pathogens were identified using the following culture media (Banhart et al., 2014; Koch-Edelmann et al., 2017; Bazin, 2018; Abdel-Wahab et al., 2022):

- Blood agar medium: this medium was used to support the growth of fastidious pathogens and to identify microbial hemolytic activity.
- MacConkey’s agar medium: this medium was used to support selective growth and differentiation of enteric gram-negative bacilli.”
- Bile esculin agar medium: this medium was used for selective growth of *Enterococcus* Spp.
- Analytical profile index: this was used for isolation of gram-negative bacilli.

#### 2.5.2. Gram staining and identification of isolated pathogens

Gram stain was used to identify microbial Gram reaction, morphology and arrangement (Abdel-Wahab et al., 2022). To identify the microorganisms, the following biochemical tests were used for microbial identification:

- Catalase production test: this test was used to differentiate *Staphylococci* from *Streptococci & Enterococci*.
- Coagulase production test: this test was used to differentiate *S. aureus* from CONS.
- Oxidase production test: this test was used to differentiate *Enterobacteriacae* from the rest of gram negative bacilli.

### 2.6. Samples processing

#### 2.6.1. Preparation of blood samples

At the conclusion of the experiment, samples were collected from the liver and then centrifuged at 3000 rpm (1,500 g) for 15 min to separate the serum. The obtained serum was immediately stored at −80°C until required for the biochemical assays. Serum liver transaminase levels, including aspartate aminotransferase (AST, product number: MAK055) and alanine aminotransferase (ALT, product number: MAK125), were measured using an automated spectrophotometric method with the Synchron cx5 autoanalyzer (Beckman, United States).

#### 2.6.2. Preparation of tissue samples

After a four-week period, rats were sacrificed, and their livers were rapidly removed. ELISA kits from MyBioSource Company (San Diego, California, USA) were utilized for analysis. The right lobe of each liver was excised for histological examination, while the other lobe was frozen at −80°C until homogenate for biomarker assessment.

### 2.7. Biochemical studies

#### 2.7.1. Assessments of liver toll like Receptor-4 (TLR4)

Assessment of Liver Toll-Like Receptor 4 (TLR4) in rats was conducted using a Rat ELISA kit (Cat.No:CSB-E15822r) for the quantitative determination of TLR4 concentrations in tissue homogenates. To prepare the tissue homogenates, 100 mg of tissue was rinsed with 1XPBS, homogenized in 1 mL of 1X PBS, and stored overnight at −20°C. After two freeze–thaw cycles were performed to break the cell membranes, the homogenates were centrifuged for 5 min at 5000 × g, 2–8°C. The supernate was removed and immediately assayed. Alternatively, the samples were stored at −80°C. and centrifuged again before the assay.

#### 2.7.2. Assessment of anti-nuclear antibody (ANA) in the liver

This assessment was conducted using a rat ELISA kit (Cat. No: MBS269217) for *in vitro* quantitative detection of ANA concentrations in tissue homogenates according to Solomon et al. (2002), in both natural and recombinant ANA. The tissue slices were washed with 0.01 M PBS and subsequently Add tissue protein extraction reagent according to proportion of 1G: 510 mL and mix them in ice water. After being blended, mixture shall be centrifuged for 10 min at 500010000 rpm. Take supernatant tested immediately or put them at −20°C (for 1–3 months) or − 80°C (for 1–3 months) for storage.

#### 2.7.3. Assessment of liver caspase 1

The Rat ELISA kit (Cat. No E-EL-R0371) was employed to quantitatively detect caspase1 in tissue homogenates. To ensure accurate results, it is important to be cautious about hemolysis as it may impact the outcome. Therefore, the tissues should be thoroughly rinsed with ice-cold PBS (0.01 M, pH = 7.4) to remove any excess blood.

The Rat ELISA kit is designed for the quantitative detection of caspase1 in serum, plasma, tissue homogenates, and other biological fluids. For tissue homogenates, it is essential to note that hemolyzed blood may affect the accuracy of the results. Therefore, to eliminate excess blood thoroughly, it is recommended to rinse the tissues with ice-cold PBS (0.01 M, pH = 7.4). The tissue pieces should be weighed and minced into smaller fragments, which will then be homogenized in PBS (the volume depends on the tissue weight, and 9 mL PBS is suitable for 1 gram of tissue pieces). Adding a protease inhibitor to the PBS is recommended. To further disrupt the cells, you can utilize either an ultrasonic cell disrupter or subject the suspension to freeze–thaw cycles. After homogenization, the samples are then centrifuged for 5 min at 5000 × g to obtain the supernatant.

#### 2.7.4. Assessment of liver anti-smooth muscle antibody (ASMA)

A Rat ELISA kit with the catalog number (DEIA-BJ2024) was used for the analysis of tissue homogenates. The tissue was prepared by rinsing the tissue in ice-cold PBS (0.02 mol/L, pH 7.0–7.2) to remove excess blood thoroughly and weighed before homogenization. The tissues were minced into small pieces and homogenized in a certain amount of PBS with a glass homogenizer on ice. The resulting suspension was subjected to ultrasonication or to two freeze–thaw cycles to further break the cell membranes. After that, the homogenates were centrifugated for 15 min at 1500 × g (or 5,000 rpm). The supernate was removed and assayed immediately or aliquot and stored samples at −80°C.

#### 2.7.5. Assessment of liver IL-18

The Rat ELISA kit (Cat. No.: E-EL-R0567) was used for assessing tissue lysate. To begin, 80 mg of tissue was washed with 1X PBS and homogenized in 1 mL of 1X PBS. Afterward, the homogenates were stored overnight at −20°C. To disrupt cell membranes, two freeze–thaw cycles were performed, followed by centrifugation for 5 min at 5000 x g and a temperature of 2–8°C. The resulting supernatant was collected and immediately used for the assay.

#### 2.7.6. Assessment of liver nod-like receptor (NLRP3)

Total RNA extraction was conducted using the RNeasy Mini Kit. The extracted RNA was then subjected to reverse transcription using the QuantiTect Reverse Transcription Kit. Real-time PCR analysis was performed using the QuantiTect SYBR Green PCR Kit. All kits utilized in the experiment were procured from Qiagen, located in Hilden, Germany. As a housekeeping gene, β-actin was employed for normalization purposes (Primers; Forward: 5′-AGGGAAATCGTGCGTGAC-3′, Reverse: 5′CGCTCATTGCCGATAGTG-3′, GenBank NM_031144.3). For NLRP3, the primers were (Forward: 5′ − CCAGGGCTCTGTTCATTG-3′, Reverse: 5′ − CCTTG GCTTTCACTTCG-3′, GenBank NM_001191642.1). In real-time PCR, a reaction mixture volume of 20 μL was prepared, consisting of 500 ng cDNA and 0.5 μM of each primer. The PCR cycling protocol consisted of an initial denaturation step at 95°C for 15 min, followed by 40 cycles. Each cycle involved denaturation at 94°C for 15 s, annealing at 55°C for 30 s, and extension at 70°C for 30 s. The relative expression of the target genes was determined using the 2^(–ΔΔCT) equation, and the results were presented as fold expression compared to a reference gene, β-actin. All kits and reagents used in the experiment were obtained from Qiagen, located in Hilden, Germany.

### 2.8. Histopathological studies

All animals were anesthetized using thiopental sodium (intraperitoneal). The right lobe of the liver of each rat was taken and cut into small pieces (1 cm^2^). The jejunum was removed, opened longitudinally and washed properly with saline to remove food remnants and mucous. One half of the liver specimen and part of the jejunum were fixed immediately in 10% buffered formaldehyde and processed to obtain paraffin blocks for light microscopic study. The other half of the liver specimen and part of the jejunum were cut into very small pieces 1 mm^2^ and fixed immediately in glutaraldehyde and prepared for transmission electron microscopic examination (TEM).

#### 2.8.1. Light microscopic assessments

Hematoxylin & Eosin (H&E) and Mallory’s trichrome stains were utilized for liver and small intestine. Caspase-8 immunohistochemical technique was applied for liver to detect the cleaved caspase-8 sections using an avidin biotin-peroxidase technique (purchased from Cell Signaling Technology, USA). Examination was done for detection of any microscopic changes using an Olympus CKX41 light microscope (Olympus Corporation, Tokyo, Japan). The reaction with a solution of DAB (purchased from DAKO, Denmark) was performed at a dilution of 1:100 for 1 hour (Horobin, 2008). Then, the counterstain was assessed by applying Mayer’s hematoxylin. The negative control sections were obtained by the same steps, except for the use of the primary antibody.

#### 2.8.2. Transmission electron microscopic examination (TEM)

The liver specimen and part of the jejunum were into small pieces of 1 mm^3^ and were rapidly fixed in 2.5% glutaraldehyde. Specimens were processed and embedded in Eponresin. Semi thin sections (50 nm) were stained with toluidine blue. Ultrathin sections were cut and mounted on copper grids (Pearce et al., 2018). Specimens were examined and photographed using a JEM 1200 EXII, JEOL, (Tokyo, Japan transmission electron microscope) at the EM unit, Faculty of Science, Ain Shams University.

#### 2.8.3. Morphometric study

Measurements were accomplished using an image analyzer Leica Q win V.3 program installed on a computer in the Histology and Cell Biology Department, Faculty of Medicine, Ain Shams University. The computer was connected to a Leica DM2500 microscope (Wetzlar, Germany). Six specimens from six different rats of each group were examined. For each specimen, six different captured non-overlapping high-power fields (×40) were taken, six different readings from every captured photo were counted and the mean was calculated for each specimen.

- Mucosal thickness, and the width of intestinal villi, measured in H&E stained intestinal sections (×20 power lens).
- Area % of collagen fibers in Mallory’s trichrome stained sections of liver and intestine (×20 power lens).
- Number of hepatocytes with positive expression of Caspase-8 in immunohistochemically stained sections (×40 power lens).

### 2.9. Clinical study

#### 2.9.1. Patients and methods

A prospective study was conducted at Ain Shams University Children’s Hospital from January 2022 to May 2022. The study obtained ethical approval from the local Ethics Committee of Ain Shams University, and written informed consent was secured from the parents of all participants. The study included one hundred and twenty male children. Control group (*n* = 60) male children aged 4 to 6 year who have ASD without AIH and history of NSAIDs intake and diseased group (*n* = 60). who met the inclusion criteria, which were being between the ages of 4 and 6 years, male gender, and having a history of receiving non-steroidal anti-inflammatory drugs (NSAIDs) during their early life, or a diagnosis of (ASD) or (AIH). Children who were older than 10 years of age or of the female gender were excluded from the study. While adhering to these criteria and securing informed consent, the study aimed to guarantee the ethical well-being of participants and acquire dependable results.

#### 2.9.2. Biochemical analysis

##### 2.9.2.1. Assessment of ANA IgG

Enzyme immunoassay for the qualitative detection of IgG anti-nuclear antibodies (ANA) in human serum/plasma was employed to test the detection of autoantibodies against intracellular antigens known as antinuclear antibodies (ANA) (CatNo.PT-ANA-SCIgG-96) from (PISHTAZ TEB DIAGNOSTICS). ANA testing is often carried out, following Jaskowski et al. (1996). When there is a strong suspicion that an underlying autoimmune disorder develops, as part of the initial diagnostic process. These antibodies are also found in patients with organ-specific autoimmune diseases (autoimmune liver diseases, Hashimoto’s thyroiditis), In this approach, microwells are coated with certain quantities of nuclear antigens. Then, the antinuclear antibodies in sample are allowed to interact with the solid phase antigens. After incubation and washing, the enzyme conjugate will be added. Following the second wash process, a chromogen-substrate solution is added and incubated for 15 min, which causes the formation of a blue color. The color development is terminated by adding a stop solution, and the color is altered to yellow and detected spectrophotometrically at 450 nm.

##### 2.9.2.2. Assessment of anti-smooth muscle antibodies (ASMA)

ASMA evaluation was conducted using a solid phase enzyme immunoassay with an INOVA Diagnostics (San Diego, CA) ELISA kit. The assay involved incubating calibrators, positive and negative controls, and diluted serum samples on antigen-coated 96-well plates for 30 min. During this incubation, the test sample’s antibodies bound to the coated wells. After the 30-min incubation, the wells were washed, and horseradish peroxidase-conjugated anti-human IgG was added. Following another 30-min incubation with the substrate, the reaction was stopped. Optical density was determined using a spectrophotometer, and a cutoff of <20 U/IU was applied. In cases of a positive result, a confirmatory test using the F-actin IgG enzyme-linked immunoassay kit from INOVA Diagnostics (San Diego, CA) was performed. In this study, a positive autoantibody screen was defined as any test with a titer of 1/20 for ASMA and 1/40 for ANA, based on the diagnostic criteria for autoimmune hepatitis from the International Autoimmune Hepatitis Group and the American Association for the Study of Liver Diseases clinical practice guidelines (Czaja and Freese, 2002).

##### 2.9.2.3. Assessment of AST, and ALT

Liver function was assessed by evaluating the activity of transaminases enzymes AST (product number: E.C.2.6.1.2), and ALT (product number: E.C.2.6.1.2) in serum, using endpoint colorimetric assay kits provided by Sigma-Aldrich Darmstadt, Germany, according to Wree et al. (2018). These enzymes are frequently tested clinically as part of a diagnostic liver function test to detect the health of the liver. Serum samples directly diluted in the assay buffer and test samples were prepared up to 20 μL/well. The tested samples numerous dosages of sample at (λ max = 570 nm) to ensure the readings are within the standard curve range.

##### 2.9.2.4. Assessment of IL 6, and IL 18

Serum IL6 and IL8 levels were measured using a human IL6, IL8 ELISA Max^™^ Set Deluxe (BioLegend, Inc., San Diego, CA, United States) following the manufacturer’s guidelines. To begin, 96-well plates were coated with the antibody one day prior to the experiment. After an 18-h incubation at 4°C, the plates were washed with PBS containing 0.05% Tween-20 (Sigma-Aldrich, St. Louis, MO, United States) and then incubated at room temperature with a diluent buffer to prevent nonspecific binding. Following rinsing, 100 mL of the sample was added to each well and incubated for 2 h at room temperature. After washing the plates again, 100 mL of biotinylated detection antibody was added to each well and incubated for 1 h. Subsequently, 100 mL of avidin-horseradish peroxidase (HRP) was added to each well and incubated for 30 min at room temperature. After another round of washing, 3,3′,5,5′-tetramethylbenzidine (TMB) substrate solution was added, and the plates were incubated in the dark for 15 min. The reaction was stopped by adding 100 mL of 2 N sulfuric acid, and the absorbance at 450 nm and 570 nm was recorded (Kawasumi et al., 2014).

##### 2.9.2.5. Assessment of NLRP3 RNA extraction and quantitative real-time PCR

For reverse transcription of RNA, the high-capacity cDNA reverse transcription kit (iScript TM) was utilized. The one-step RT-PCR Kit with SYBR^®^ Green from BioRad, located in Hercules, CA, United States, was employed for this purpose. The primers used for amplifying the target gene were as follows: NLRP3 forward 5′-GCA GCA AAC TGG AAA GGA AG-3′ and reverse 5′-CTT CTC TGA TGA GGC CCA AG-3′; ASC forward 5′-GCACTT TAT AGA CCA GCA CCG-3′ and reverse 5′-GGC TGG TGT GAA ACTGAA GA-3′. Quantitative real-time PCR was performed using the LightCycler CFX96 system (BioRad, Hercules, CA, United States) following the manufacturer’s instructions. To achieve optimal results, primer concentration was titrated within the range of 100 to 500 nM, and a final concentration of 300 nM per primer was found to be effective for most reactions. Equal concentrations of each primer were used to optimize the reaction efficiency. The amplicon size was limited to a range of 50 to 200 bp. Relative mRNA expression levels were determined using the comparative 2^(−ΔΔCT) method (Lebuhn et al., 2016).

##### 2.9.2.6. Assessment of JAK-1 utilizing ASO specific-PCR genetic marker

To detect the JAK1 mutation, the following primers were used: (JAK1 Reverse: 5′CTGAAT AGTCCTACAGTGTTTTCAGTTTCA 3′, JAK1 Forward (specific): 5′ AGCATTTGGTTTTAAATTATG GAGTATATT 3′ and JAK1 Forward (internal control): 5′ ATCTATAGTCATGCTGAAAGTAGGAGAAAG 3′). The PCR amplification consisted of 35 cycles with an annealing temperature of 58.5°C. Subsequently, agarose gel electrophoresis (2%) was performed to visualize and detect the resulting DNA bands (Baxter et al., 2005).

### 2.10. Statistical analysis

In this study, all data were expressed as means±SD, providing a measure of the variability within the dataset. To analyze the data, we utilized GraphPad Prism software program, version 5.0 (2007) from GraphPad Software, Inc., based in CA, USA. The statistical comparison among different groups was carried out using the Unpaired *T*-test, a widely used method to assess differences between two independent groups. For further comparison between these groups, we applied a *post hoc* “Transform Normalized Test. Statistical significance was set at *p* < 0.05, indicating that any differences observed with this level of probability were considered statistically significant. Additionally, we performed an AUROC analysis using the SPSS software package, version 20, from SPSS Inc., headquartered in Chicago, IL, USA. This analysis allowed us to assess the diagnostic accuracy of the measurements and evaluate the potential of the variables in distinguishing between different groups. The determination of the sample size was accomplished using GraphPad Stat Mate software program, Version 1.01, from January 16, 1998. This step ensured that our study had an appropriate sample size to provide statistically meaningful results and reliable conclusions.

### 2.11. Ethical considerations

Ethical conduct in experimenting on laboratory animals was carefully considered throughout the study. To ensure ethical conduct, the plausibility of the study questions was ascertained through a literature review. Choosing the animal model was based on an accepted level of consensus across the thoroughly searched literature. Sample size was kept at the minimum required to provide the power sufficient for statistical comparisons. Housing conditions (e.g., illumination, temperature, noise, cleanliness, standard diet and number of animals/cage) were monitored and adequately maintained. Handling animals, for injections or other treatments, was dispensed with the minimal stress possible. Euthanasia was strictly adhered to such that sacrificing animals was performed under appropriate anesthesia. All animals underwent intraperitoneal anesthesia using thiopental sodium at a dose of 100 mg/kg. Thiopental sodium is a barbiturate drug commonly used for induction and maintenance of anesthesia in laboratory animals. The choice of thiopental sodium as the anesthetic agent is likely due to its rapid onset of action and relatively short duration of effect, allowing for smooth and controlled anesthesia induction (Mohamed et al., 2020). Samples obtained from the sacrificed animals was carefully analyzed using the most robust analytical techniques available, so as to maximize the benefit out of each sacrificed animal. Regarding the clinical study (human study), written informed consent was obtained from all participants, and the study was conducted in accordance with the Declaration of Helsinki and approved by the Ethics Committee. Additionally, it received approval from the Ethics Committee of the Ain Shams Faculty of Medicine, Egypt (No. FWA 00017585, 08/2023) (Kalinichenko et al., 2021; Mohamed et al., 2021, 2022a,b).

## 3. Results

### 3.1. Assessment of bacterial translocation

We initially assessed the effect of indomethacin administration on the bacterial translocation in the blood culture. As shown in Table 1, the evaluation of blood culture in rats before indomethacin administration (control group) revealed a negative bacterial translocation in both juvenile and adult groups. On the other hand, administration of indomethacin (3 mg/kg/day, for 4 weeks) in juvenile wistar rats demonstrated a positive bacterial translocation in the blood culture of 80% of the group, as compared to the control group, including 30% *E. coli*, 30% *Enterococcus* Spp., and 20% *Klebsiella* Spp. Similarly, indomethacin administration in adult wistar rats displayed a positive bacterial translocation in the blood culture of 60% of the group (40% *E. coli*, 20% *Enterococcus* Spp.). These results indicate that administration of indomethacin induces intestinal permeability which leads to bacterial translocation (dysbiosis). Notably, the effect of indomethacin uptake in bacterial translocation was more significant in juvenile group more than that in adult group.

**Table 1:** Effect of indomethacin administration (3 mg/kg/day) for 4 weeks on bacterial translocation.

	Adult		Juvenile	
	Control	Indomethacin group	Control	Indomethacin group
Positive blood culture	Zero	60%	Zero	80%
Negative blood culture	10	3 (30%) – one died case	10	1 (10%) – one died case
*E. coli* (Gram negative bacilli)	Zero	40%	Zero	30%
*Enterococcus* Spp. (Gram positive cocci)	Zero	20%	Zero	30%
*Klebsiella* Spp. (Gram negative bacilli)	Zero	Zero	Zero	20%

### 3.2. Biochemical analysis

#### 3.2.1. Effect of indomethacin administration on the serum ALT and AST levels

**Figure 1:**
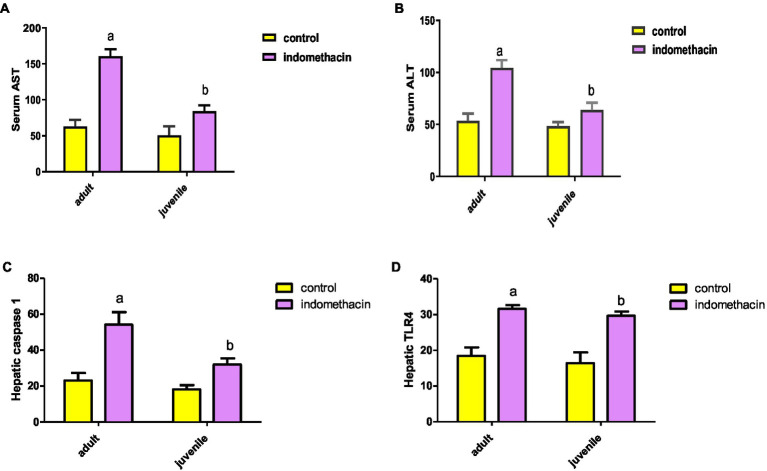
Effect of oral administration of indomethacin (3 mg/kg, 4 weeks) on the serum AST **(A)**, ALT **(B)** levels, hepatic caspase 1 expression (pg/g tissue) **(C)**, and expression of hepatic TLR4 gene **(D)** in the adult and juvenile groups. Data are presented as mean ± SD using Unpaired *T*-test, *n* = 6. ^a^ significant difference in comparison to adult control; ^a^ (*p* < 0.05), ^b^ significant difference in comparison to juvenile control; ^b^ (*p* < 0.05).

Frist, we have explored the effect of indomethacin administration (3 mg/kg/day, 4 weeks) on liver function by evaluating (alanine transaminase) ALT and AST levels in serum. As shown in Figure 1, oral administration of indomethacin significantly (*p* < 0.05) elevated the levels of serum ALT in both the juvenile group (51.00 U/L to 68.83 U/L), and the adult group (58.33 U/L to 109.5 U/L), as compared to the control groups. Similarly, administration of indomethacin demonstrated the ability to significantly (*p* < 0.05) increase of the AST levels in serum of juvenile (89.83 U/L), and adult (153.7 U/L) wistar treated rats, as compared to untreated groups (59.5 U/L and 69.50 U/L, respectively). These results reveal that NSAIDs administration (indomethacin) has deterioration effects on the liver function in both juvenile and adult subjects which range from asymptomatic elevations in serum aminotransferase levels to fulminant liver failure. These effects could be attributed to the direct hepatotoxic effect of NSAIDs or to their ability to disturb the gut microbiota and increase intestinal permeability that commonly associated with systemic release of inflammatory cytokines leading to liver cell injury.

#### 3.2.2. Effect of indomethacin administration on the expression of hepatic TLR4 gene

To explore the effect of indomethacin administration on disturbing the intestinal barrier and induction of inflammatory reaction in liver tissue, the expression of hepatic TLR4 gene was assessed by ELISA. The results demonstrated that the untreated juvenile and adult groups exhibit normal expression levels of TLR4 gene (14.23 pg./g tissue, and 16.70 pg./g tissue, respectively) (Figure 1). On the other hand, administration of indomethacin (3 mg/kg/day, for 4 weeks) significantly (*p* < 0.05) elevated the expression of hepatic TLR4 gene in both juvenile (28.77 pg./g tissue) and adult (30.83 pg./g tissue) groups. These findings suggest that indomethacin administration leads to impairment of the mucosal barrier leading to bacterial translocation. Furthermore, the increased expression of hepatic TLR4 gene triggers the inflammatory pathway by interacting with lipopolysaccharide (LPS), derived from gram-negative bacteria in the intestinal lumen, leading to the activation of the NLRP3 and IL-18 pathways.

#### 3.2.3. Effect of indomethacin administration on the expression of hepatic caspase 1

To examine the effect of indomethacin-induced intestinal dysbiosis on the inflammatory activation pathway, we have assessed the expression of hepatic caspase 1 levels by ELISA in both juvenile and adult treated groups in comparison to control group. As displayed in Figure 1, daily oral administration of indomethacin (3 mg/kg) for 4 weeks significantly (*p* < 0.05) increased hepatic caspase 1 levels in indomethacin-treated juvenile (34.4 pg./g tissue) and adult (59.08 ± 1.67 pg./g tissue) groups, as compared to the control groups (19.82 pg./g tissue, and 26.08 pg./g tissue, respectively). These findings highlight the possible effect of indomethacin-induced dysbiosis in activating NLRP3 inflammasome pathway, as caspase 1 is downstream component of NLRP3 protein complex. NLPR3 plays a crucial role to convert caspase-1 to its cleaved caspase-1 activated form, which largely triggers a series of inflammatory mediators, including interleukin (IL)-1β, IL-18, resulting in liver injury.

#### 3.2.4. Effect of indomethacin uptake on the expression of hepatic IL-18

**Figure 2:**
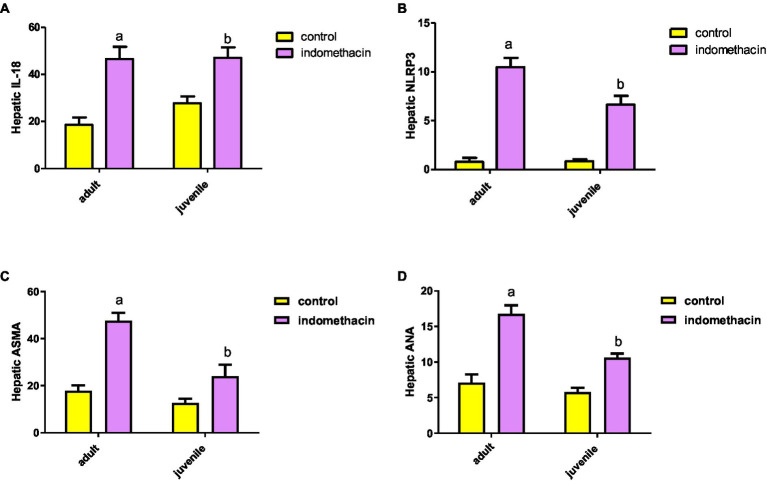
Effect of daily indomethacin administration (3 mg/kg) for 4 weeks on hepatic IL-18 expression (pg/g tissue) **(A)**, NLRP3 expression **(B)**, anti smooth muscle antibody (ASMA) **(C)**, and antinuclear antibody (ANA) **(D)** in the adult and juvenile groups. Data are presented as mean ± SD, using Unpaired *T*-test, *n* = 6. ^a^ significant difference in comparison to adult control; ^a^ (*p* < 0.05), ^b^ significant difference in comparison to juvenile control; ^b^ (*p* < 0.05).

To further explore the effect of indomethacin administration on the activation of NLRP3 inflammasome pathway, we have evaluated the expression of IL-18 by ELISA analysis. Administration of indomethacin (3 mg/kg/day, 4 weeks) induced a significant (*p* < 0.05) increase in the expression of IL-18 levels in liver tissue of juvenile (29.27 pg./g tissue) and adult (50.20 pg./g tissue) treated groups, as compared to untreated groups (17.8 pg./g tissue, and 20.8 pg./g tissue, respectively). These results demonstrated the effect of indomethacin administration on inducing NLRP3 inflammasome pathway after disturbing gut permeability. Notably the effect of indomethacin uptake on hepatic IL-18 expression was more tremendous in the adult treated group (Figure 2). Inflammasomes are signaling platforms that are activated in response to infectious diseases or chronic sterile inflammation and induce inflammation via triggering IL-1β and IL-18 or inducing pyroptosis via gasdermin-d (GSDMD). Our findings are in agreement with the observed over-expression in hepatic caspase 1 levels for indomethacin-treated groups and affirm the effect of indomethacin administration on activating NLRP3 inflammasome pathway.

#### 3.2.5. Effect of indomethacin-induced dysbiosis on hepatic NLRP3 expression

To emphasize the effect of indomethacin-induced dysbiosis on inflammasome pathway, we have examined the expression of hepatic NLRP3 gene by real-time PCR. According to the data presented in Figure 2, the juvenile and adult groups that did not receive treatment demonstrated a normal level of NLRP3 inflammasome expression (0.99 and 1.07, respectively). Interestingly, administration of indomethacin (3 mg/kg/day, 4 weeks) demonstrated the ability to significantly (*p* < 0.05) upregulate the expression of hepatic NLRP3 in both juvenile and adult treated groups (7.28 and 11.15, respectively). The NLRP3 inflammasome is the most widely studied and the best-characterized inflammasome in chronic inflammatory diseases. The NLRP3 inflammasome is composed of three main components: the NOD-like receptor NLRP3, the adaptor protein ASC (apoptosis-associated speck-like protein containing a CARD), and the effector molecule pro-caspase-1. These components work together to activate the inflammasome complex and initiate the inflammatory response. The NLRP3 inflammasome can be activated by several stimuli including pathogen-associated molecular patterns (PAMPs) ligands and damage-associated molecular patterns (DAMPs), which could be released in systemic circulation after indomethacin-induced disturbance of the intestinal permeability. NLRP3 inflammasome activation was associated with downstream activation of caspase 1, IL-1B and IL-18 leading to liver cell pyroptosis. Activation of NLRP3 inflammasome by PAMPs and DAMPs indicates that the NLRP3 inflammasome is a common sensor of cellular stress or injury. Overall, our results further highlight the pivotal role of NLRP3 inflammasome pathway in the development of liver disease.

#### 3.2.6. Effect of indomethacin administration on the expression of hepatic anti-smooth muscle antibody (ASMA) and antinuclear antibody (ANA)

To gain further insights into the molecular effect of indomethacin-induced dysbiosis on autoimmune hepatitis, we aimed to assess the expression of hepatic ASMA and ANA by ELISA analysis. As displayed in Figure 2, the untreated control groups demonstrated a normal level of hepatic antibody ASMA and ANA in both juvenile (13.95 μg/g tissue and 6.2 μg/g tissue, respectively) and adult (19.45 μg/g tissue and 7.92 μg/g tissue, respectively). On the other hand, administration of indomethacin (3 mg/kg/day, 4 weeks) significantly (*p* < 0.05) elevated the level of ASMA in the liver tissue of juvenile group (27.47 μg/g tissue) and adult group (50 μg/g tissue). Further, the indomethacin-treated groups displayed significantly (*p* < 0.05) excessive levels of ANA in both juvenile and adult groups (11.02 μg/g tissue, and 17.62 μg/g tissue, respectively). Numerous studies have provided substantial evidence regarding the association of ANA and ASMA with autoimmune hepatitis. ANA comprises antibodies that exhibit reactivity specifically directed against nuclear membranes and DNA. In contrast, the target antigens of ASMA are believed to be directed towards action, tubulin, or the intermediate filaments present in the cell. These antibody markers serve as important diagnostic tools in identifying and understanding this autoimmune liver disease. Therefore, our findings indicate the possible impact of indomethacin-induced dysbiosis in inducing autoimmune hepatitis.

### 3.3. Histological analysis

#### 3.3.1. Light microscopic analysis (H&E stain)

##### 3.3.1.1. Assessment of intestinal mucosa tissue

To assess the impact of indomethacin administration on the intestinal tissue architecture and inflammatory response in juvenile and adult groups, we employed Hematoxylin and Eosin (H&E) staining. The light microscopic analysis was conducted to examine several histological parameters including the integrity of the intestinal epithelium, underlying connective tissue layers, smooth muscle layer, villus morphology, and extent of inflammatory cell infiltration.

###### 3.3.1.1.1. Juvenile group

**Figure 3:**
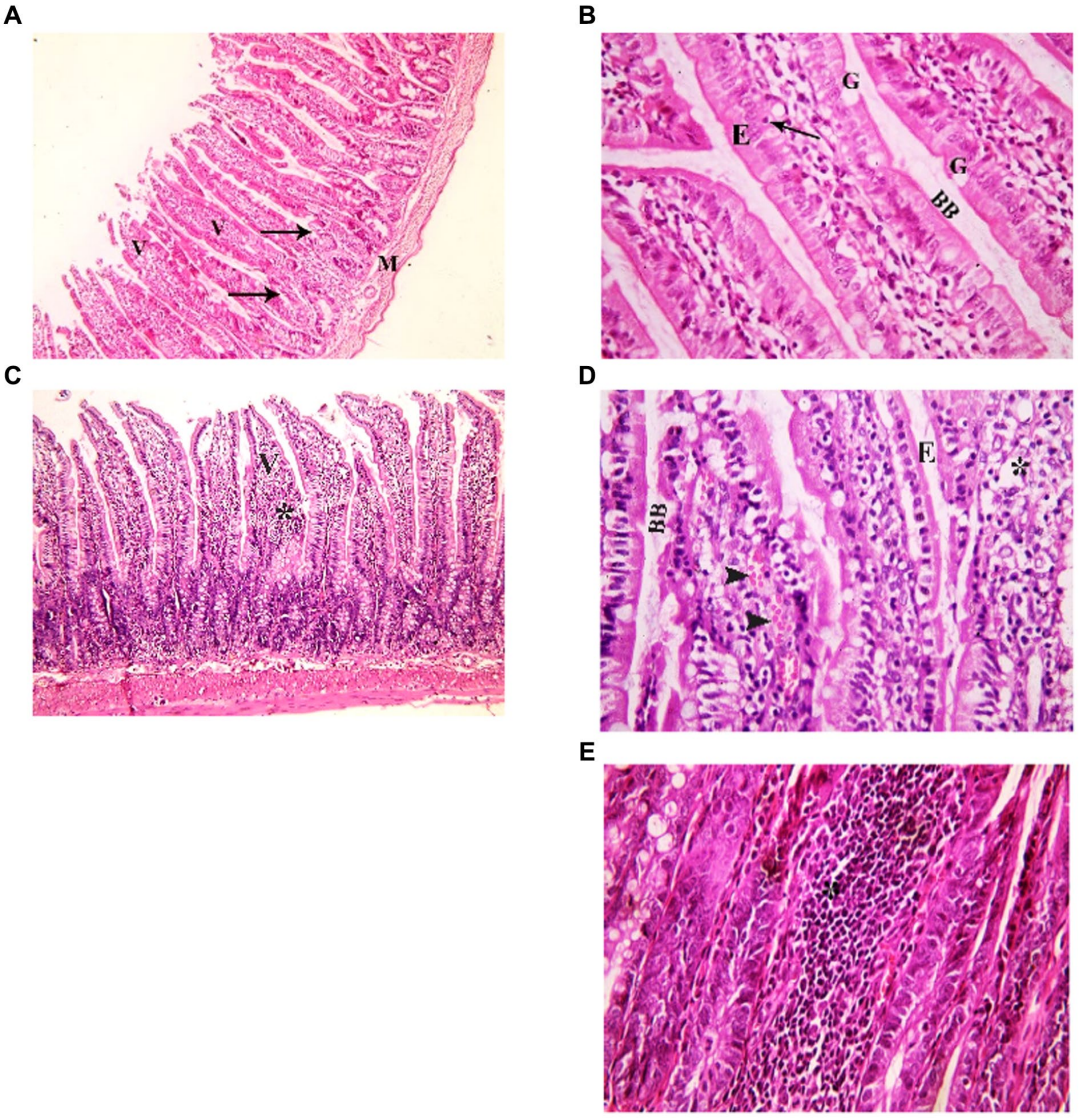
Showing intestinal mucosa in juvenile groups. **(A,B)** Control. **(A)** Showing intestinal (juvenile) mucosa formed of thin finger-like villi (V) and crypts of Lieberkühn (↑). Musculosa (M) is seen under the mucosa. **(B)** Showing enterocytes (E) columnar with acidophilic cytoplasm, oval basal vesicular nuclei and intact brush border (BB). Notice the goblet cells (G) and intraepithelial lymphocytes (↑). The core of the villi is formed of CT lamina propria with mononuclear cells. **(C–E)** Indomethacin (Model) group. **(C)** Showing distended villi (V) with apparently increased mononuclear inflammatory cells in their cores (*). **(D)** Many enterocytes (E) appear cubical with rounded deeply stained nuclei. Some villi have interrupted brush border (BB). The core of the villi is markedly vacuolated (*) and shows areas of hemorrhage (▴). **(E)** Some villi showed extensive mononuclear inflammatory cellular infiltration in their cores (*). *H&E: ***(A,C)*** × 100, ***(B,D,E)*** × 400.*

**Figure 4:**
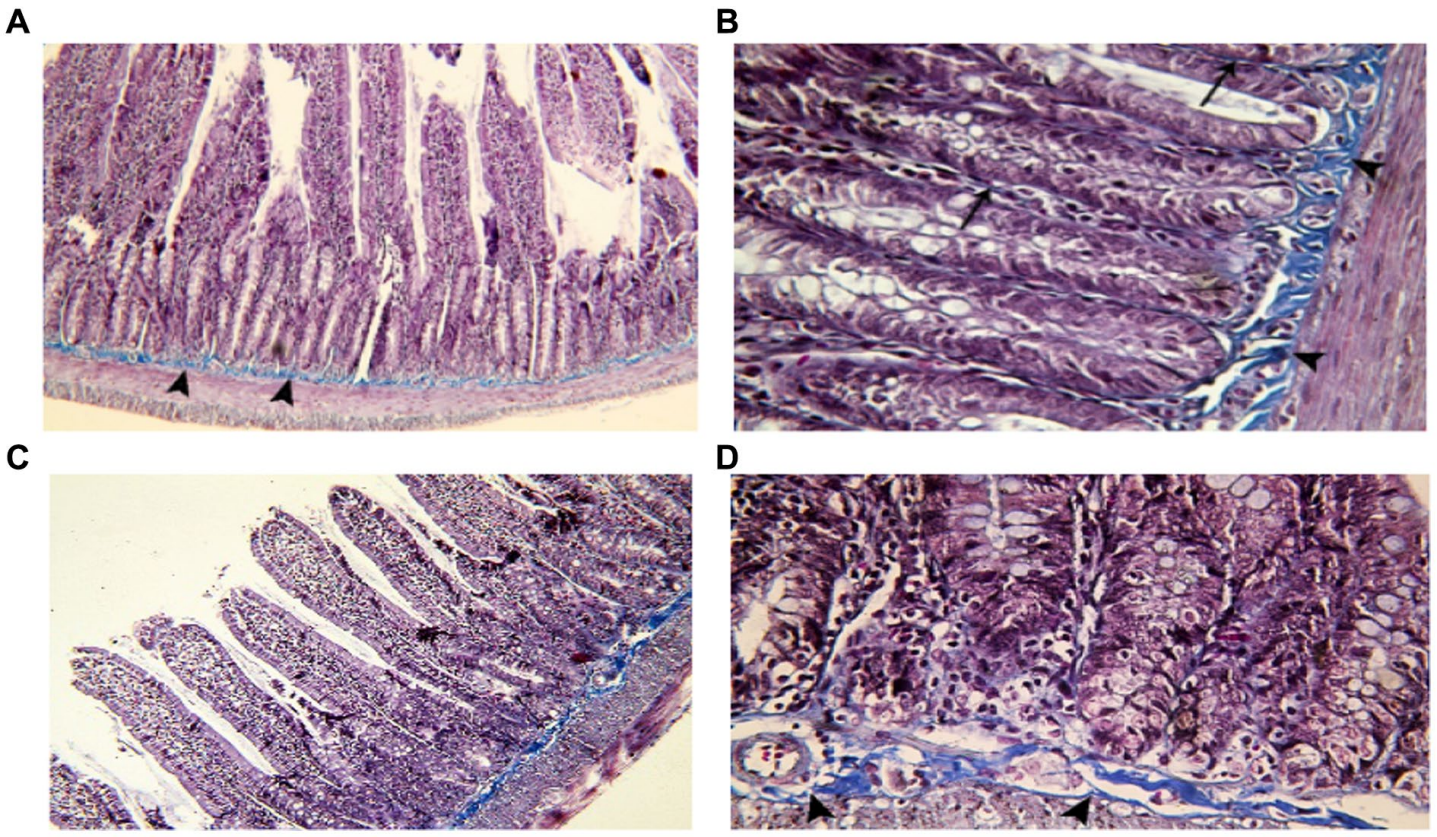
Showing intestinal mucosa in juvenile groups. **(A,B)** Control, showing continuous layer of collagen fibers (▴) separating the mucosa of the jejunum from the musculosa and few thin fibers extending into the cores of the villi (↑). **(C,D)** Indomethacin (Model) group, showing discontinuous and irregular collagen fibers (▴). *Mallory’s trichrome stain: ***(A,C)*** × 100*, ***(B,D)*** *× 400.*

**Figure 5:**
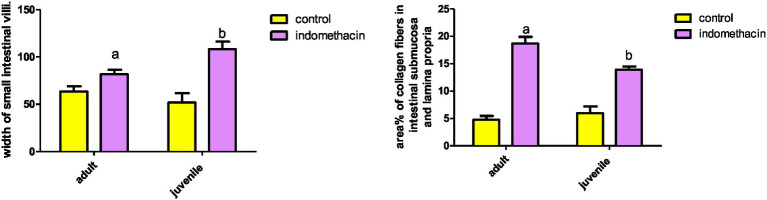
Effect of daily oral administration of indomethacin (3 mg/kg/day) for 4 weeks on mucosal thickness and width of small intestine. Data are presented as mean ± SD, *n* = 6.* significant difference in comparison to adult control; * (*p* < 0.05), # significant difference in comparison to juvenile control; # (*p* < 0.05), using Unpaired *T*-test.

Examination of H&E stained juvenile sections of the juvenile control group showed the layers of the intestinal wall; the mucosa has thin finger-like projections (villi) alternating with deep invaginations (crypts of Lieberkühn) (Figure 3). The mucosal thickness and the width of the intestinal villi measured 353.7 ± 15.46 and 58.84 ± 2.76, respectively (Figure 4). A thin connective tissue layer (submucosa) separates the mucosa from the underlying smooth muscle layer (Musculosa). The villi were seen covered with enterocytes and goblet cells; enterocytes are columnar cells with acidophilic cytoplasm, oval basal vesicular nuclei and intact brush border. Goblet cells are pale rounded cells with foamy cytoplasm. Intraepithelial lymphocytes were also seen. The core of the villi is formed of CT lamina propria with mononuclear cells examination of H&E stained juvenile sections of the indomethacin-treated juvenile model group showed the villi distended with apparently increased mononuclear inflammatory cells in their cores. Many enterocytes appeared cubical with rounded deeply stained nuclei. Some villi had interrupted brush border. The core of the villi was markedly vacuolated. Some villi showed extensive mononuclear inflammatory cellular infiltration in their cores. The mucosal thickness and the width of intestinal villi measured 713.0 ± 8.83 and 113.8 ± 4.69, respectively (Figure 5). These histological observations reveal that the administration of indomethacin resulted in a reduction in the thickness of intestinal layers, accompanied by an enlargement of the intestinal villi. Additionally, these changes were associated with an increased infiltration of inflammatory cells within the mucosal tissue. These results affirm the effect of indomethacin in the impairment of the intestinal wall permeability and induction of dysbiosis.

###### 3.3.1.1.2. Adult group

**Figure 6:**
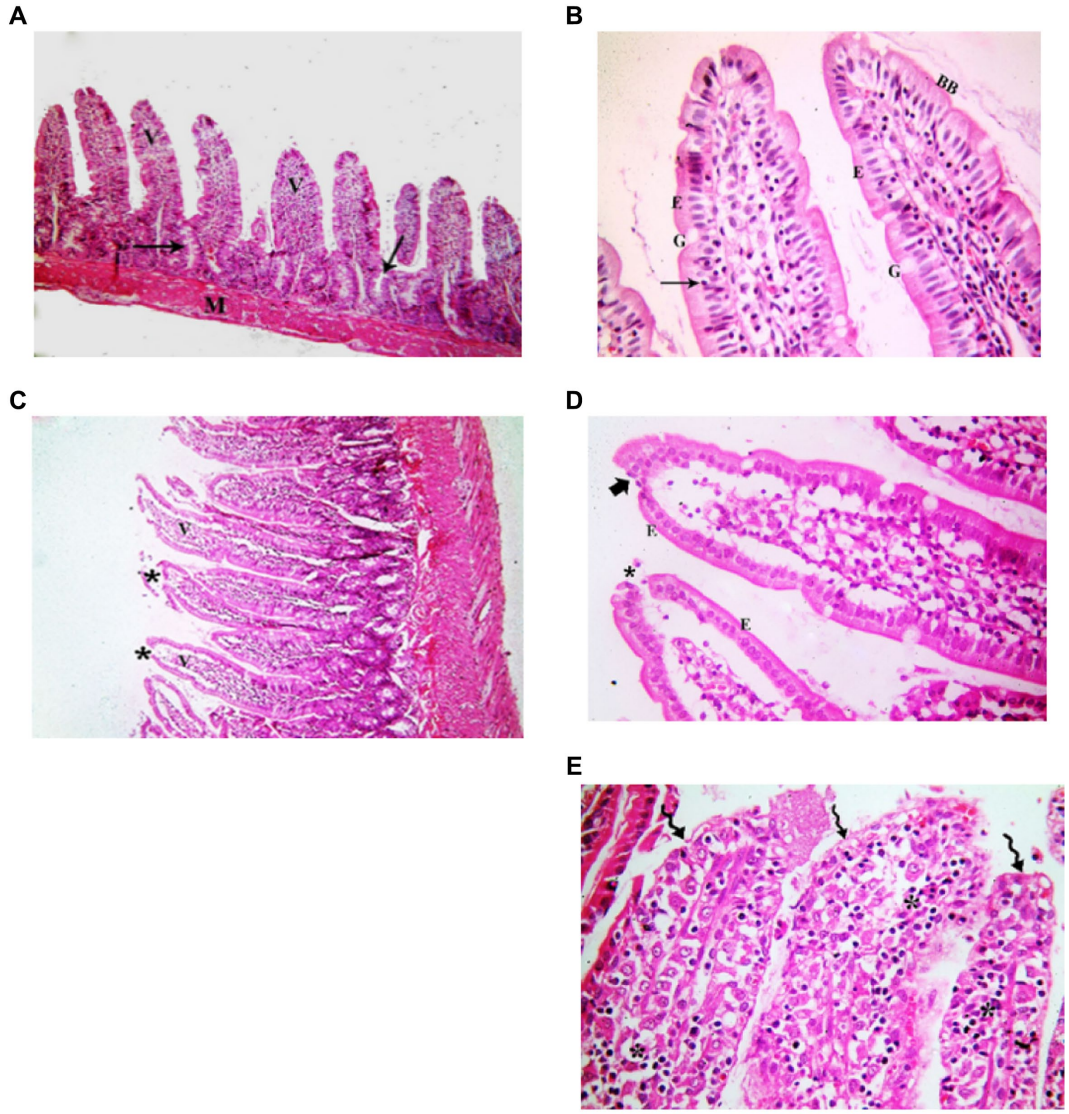
Showing intestinal mucosa in adult groups. **(A,B)** Control. **(A)** Intestinal (Jejunal) mucosa with finger-like villi (V) and crypts of Lieberkühn (↑). Musculosa (M) is seen under the mucosa. **(B)** Enterocytes (E) are columnar with acidophilic cytoplasm, oval basal nuclei and intact brush border (BB). Notice the goblet cells (G) and intraepithelial lymphocytes (↑). The core of the villi is formed of CT lamina propria with mononuclear cells. **(C–E)** Indomethacin (Model) group. **(C)** Villi (V) are seen swollen and distorted. Some villi have sloughed tips (*). **(D)** Most of the enterocytes (E) appear cubical with rounded nuclei. Some villi have sloughed tips (*) and others have areas of epithelial proliferation (thick arrow). The core of the villi is markedly vacuolated. **(E)** Some villi showed sloughing of their epithelium (waved arrows) and increased number of mononuclear inflammatory cells in their cores (*). *H&E: ***(A,C)*** × 100. ***(B,D,E)*** × 400.*

Assessment of H&E stained jejunal sections of the adult control group showed the normal layers of the intestinal wall (Figure 6). The mucosal thickness and the width of the villi measured 350.9 ± 6.59 and 67.37 ± 1.42, respectively (Figure 5). Examination of H&E stained jeunal sections of the indomethacin-treated adult model group showed the villi swollen and distorted. Most of enterocytes appeared cubical with rounded deeply stained nuclei Some villi had sloughed tips while others had areas of epithelial proliferation. The cores of the villi were markedly vacuolated. Some villi showed sloughing of their epithelium and extensive mononuclear inflammatory cellular infiltration in their cores. The mucosal thickness and the width of the villi measured 476.3 ± 12.50 and 85.08 ± 2.97, respectively (Figure 5). Based on the aforementioned histological findings, it can be concluded that the administration of indomethacin induced similar histological effects as observed in the juvenile group. Notably, the effects were more pronounced in the juvenile group compared to adult group.

##### 3.3.1.2. Assessment of liver tissue

Next, we assessed the effect of indomethacin administration on the liver tissue parenchyma of juvenile and adult groups by conducting H&E stain. The light microscope analysis was employed to examine the degree of inflammatory cell infiltration and hepatocyte structure.

###### 3.3.1.2.1. Juvenile group

**Figure 7:**
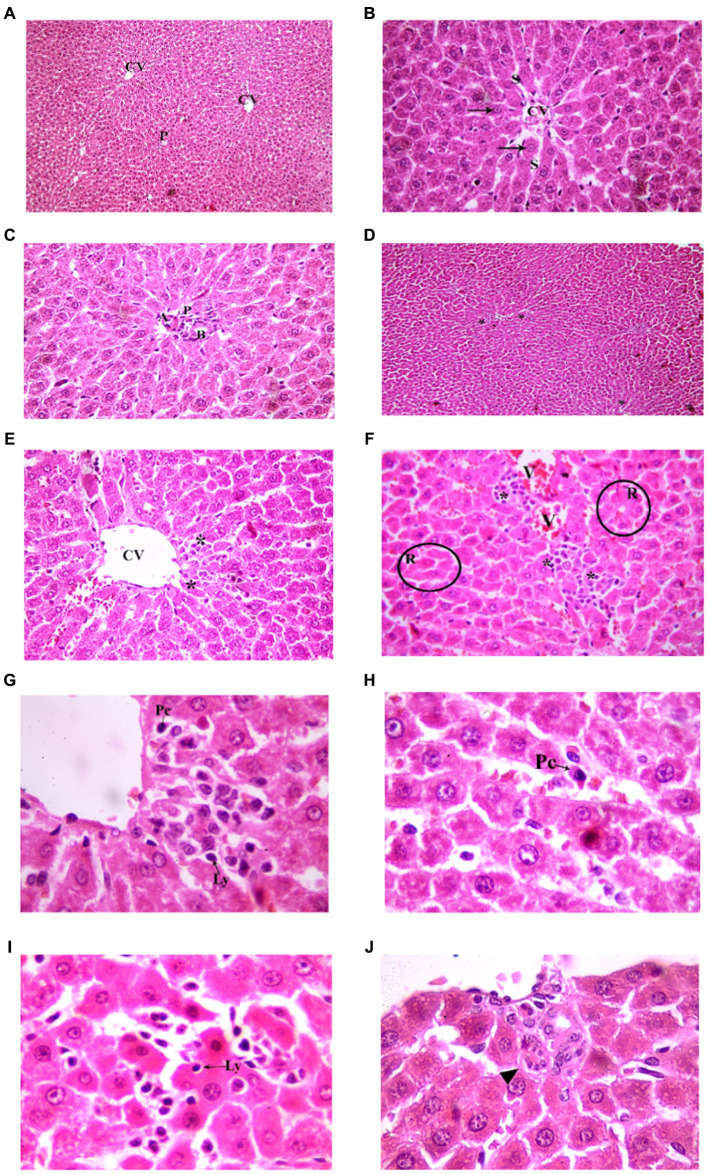
Showing liver sections in juvenile groups. **(A–C)** Control. **(A)** Normal hepatic architecture with Central veins (CV) and a portal area (P). **(B)** Showing a central vein (CV) with radiating cords of hepatocyte (↑), separated by blood sinusoids (S). **(C)** Showing portal area with a branch of bile duct (B), hepatic artery (A) and portal vein (P). **(D–J)** Indomethacin (Model) group. **(D,E)** showing mononuclear inflammatory cells (*) in-between hepatocytes and near a central vein (CV). **(F)** some hepatocytes form rosettes (R), mononuclear inflammatory cell infiltrate (*) is seen around a congested vessel (V) and extending between hepatocytes. **(G)** The inflammatory infiltrate is formed mainly of lymphocytes (Ly) and plasma cells (Pc). **(H)** A plasma cell (Pc); oval, basophilic with rounded eccentric deeply stained nucleus. **(I)** A lymphocyte (Ly) is seen inside a hepatocyte (Emperipolesis). **(J)** A multinucleated giant cell (▴). *H&E: ***(A,D)*** × 100; ***(B,C,E,F)*** × 400; ***(G,H,I,J)*** × 1,000.*

Examination of H&E stained liver sections of the juvenile control group showed the normal architecture of the liver parenchyma in the form of polygonal lobules with central veins in their centers and portal areas at the corners. From the central veins, there were radiating cords of polygonal, acidophilic hepatocytes with central vesicular nuclei. They were separated by blood sinusoids. The portal areas contained branches of bile duct, hepatic artery and portal vein (Figure 7). Examination of H&E stained liver sections of the juvenile model group showed diffuse mononuclear inflammatory cells within the hepatic lobules. Mononuclear inflammatory cells were seen near the central veins, portal areas and extending in-between the hepatocytes (interface hepatitis). The inflammatory infiltrate is formed mainly of lymphocytes and plasma cells. Plasma cells appeared oval with basophilic cytoplasm and rounded eccentric deeply stained nucleus. Some groups of hepatocytes were seen arranged around a lumen forming rosettes. Sometimes intact lymphocytes were seen inside hepatocytes (Emperipolesis). Also multinucleated giant cells were sometimes seen (Figure 7). The histological findings presented above reveal the presence of lymphocytes and plasma cells, which are recognized histological markers of autoimmune hepatitis, within the liver tissue parenchyma of both the juvenile and adult groups treated with indomethacin. These observations provide compelling evidence supporting the role of indomethacin administration in triggering autoimmune hepatitis.

###### 3.3.1.2.2. Adult group

**Figure 8:**
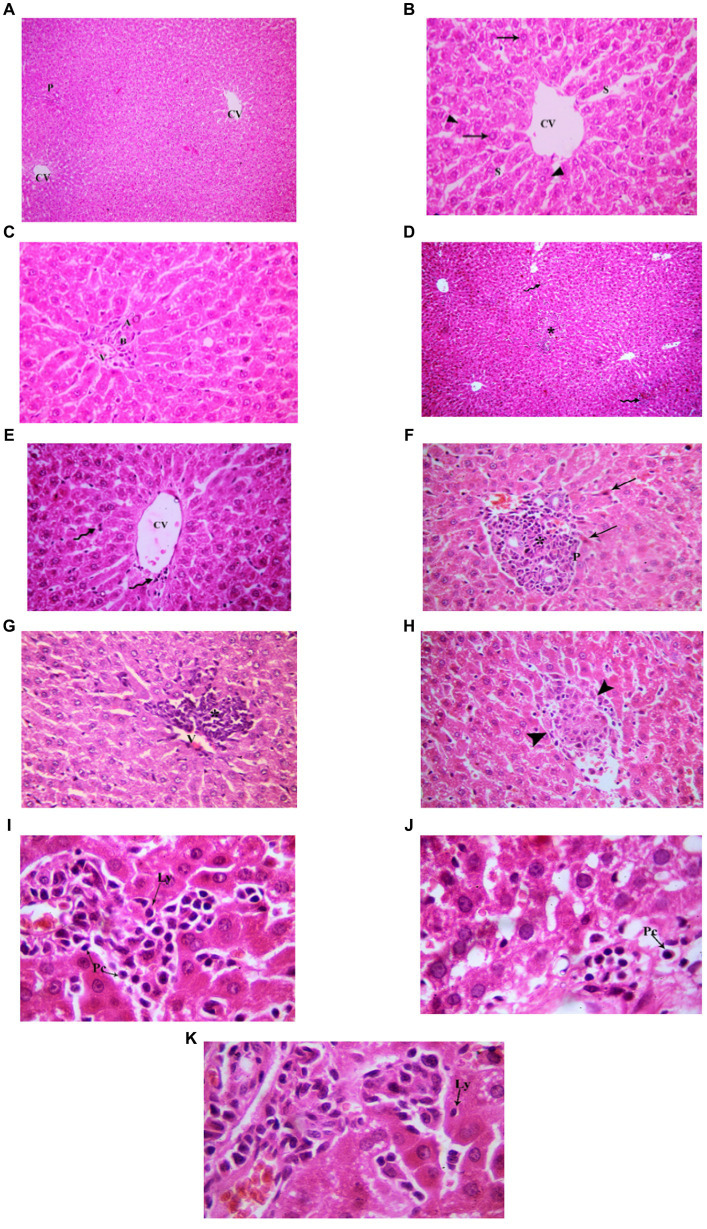
Showing liver sections in adult groups. **(A–C)** Control. **(A)** Showing normal hepatic architecture with central veins (CV) and a portal area (P). **(B)** Showing cords of hepatocytes (↑) separated by blood sinusoids (S). Some hepatocytes are binucleated (▴). **(C)** Showing branch of bile duct (B), hepatic artery (A) and portal vein (P). **(D–K)** Indomethacin group. **(D)** Showing diffuse (waved arrow) and aggregated (*) mononuclear inflammatory cells. **(E)** showing mononuclear inflammatory cells (waved arrows) near the central vein (CV) and in-between the hepatocytes. **(F)** A portal area (P) with mononuclear inflammatory cell infiltrate (*). Some hepatocytes (↑) are shrunken with pyknotic nuclei and deeply stained acidophilic cytoplasm. **(G)** Mononuclear inflammatory cell infiltrate (*) is seen around a central vein (V) and extending between hepatocytes. **(H)** Area of focal necrosis (▴) within a hepatic lobule. **(I)** Inflammatory infiltrate formed mainly of lymphocytes (Ly) and plasma cells (Pc). **(J)** Showing a plasma cell (Pc). **(K)** A lymphocyte (Ly) is seen inside a hepatocyte (Emperipolesis). *H&E: ***(A,D)*** × 100; ***(B,C,E,F,G,H)*** × 400; ***(I,J,K)*** × 1,000.*

Examination of H&E stained liver sections of the adult control group showed the normal architecture of the liver parenchyma in the form of polygonal lobules with central veins in their centers and portal areas at the corners (Figure 8). Assessment of H&E stained liver sections of the treated-adult model group showed diffuse mononuclear inflammatory cells within the hepatic lobules. Mononuclear inflammatory cells were seen near the central veins, with different amounts ranging from only few cells to large areas of aggregated mononuclear inflammatory cells, extending between the hepatocytes. Also, the portal areas showed mononuclear inflammatory cell infiltrate (interface hepatitis). The inflammatory infiltrate was formed mainly of lymphocytes and plasma cells. Plasma cells appeared oval with basophilic cytoplasm and rounded eccentric deeply stained nucleus. Sometimes intact lymphocytes were seen inside hepatocytes (Emperipolesis). Some hepatocytes appeared shrunken with pyknotic nuclei and deeply stained acidophilic cytoplasm. Also, necrotic areas with homogenous acidophilic centers and inflammatory cells were seen (focal necrosis) (Figure 8). Based on the aforementioned histological findings, it can be concluded that the administration of indomethacin resulted in similar histological effects as observed in the juvenile group. Notably, the impact of indomethacin was more pronounced in the juvenile group compared to other groups.

#### 3.3.2. Light microscopic analysis (Mallory’s trichrome stain)

##### 3.3.2.1. Assessment of intestinal mucosa tissue

To gain insight into the degree of the inflammatory reaction in intestinal fibrous tissue, we employed Mallory’s Trichrome Stain to assess the amount of collagen tissue deposition in intestinal wall layers of juvenile and adult groups.

###### 3.3.2.1.1. Juvenile group

Examination of Mallory’s trichrome stained jejunal sections of the juvenile control group showed a layer of regularly arranged collagen fibers between the mucosa and the musculosa with few thin fibers extending into the cores of the villi. The area % of collagen fibers in the submucosa and lamina propria measured 6.83 ± 0.933 (Figure 4). Examination of Mallory’s trichrome stained juvenile sections of the juvenile model group showed apparently increased amount of irregularly arranged collagen fibers between the mucosa and the musculosa. The area % of collagen fibers in the submucosa and lamina propria measured 14.32 ± 1.16. The results suggest that the use of indomethacin leads to an increase in the deposition of collagen fibers in the juvenile section when compared to the control group. This increase may be attributed to the inflammatory response triggered by the administration of indomethacin.

###### 3.3.2.1.2. Adult group

**Figure 9:**
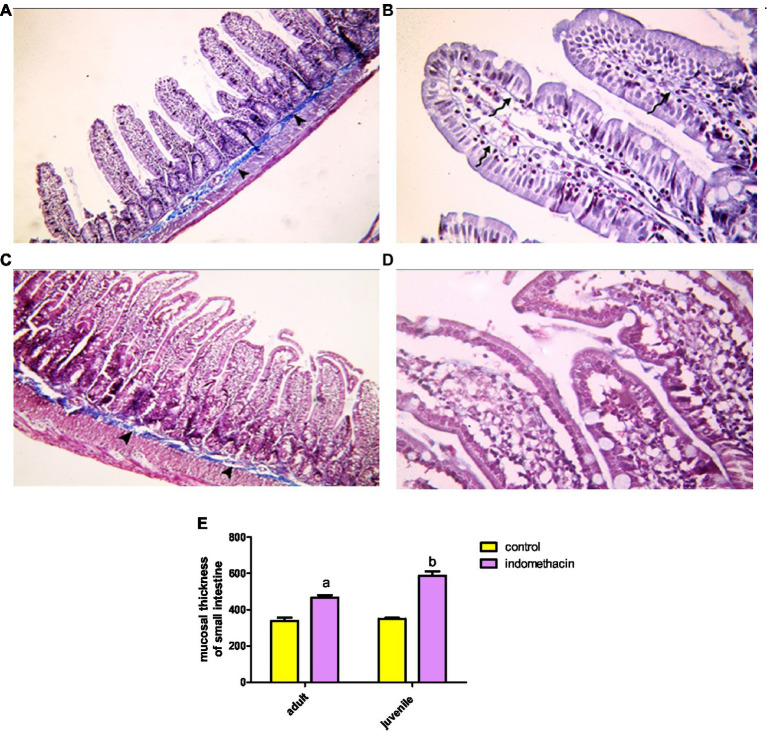
Showing intestinal mucosa in adult groups. **(A,B)** Control. **(A)**: showing the mucosa of the jejunum separated from the musculosa by a layer of collagen fibers (▴). **(B)**: showing thin, longitudinal collagen fibers inside the villi (waved arrow). **(C,D)**: indomethacin (Model) group. **(C)**: the collagen fibers appear discontinuous and irregular (▴). **(D)**: most of the villi are seen with distorted collagen fibers. *Mallory’s trichrome stain: ***(A,C)*** × 100; ***(B,D)*** × 400*. **(E)**: effect of daily oral administration of indomethacin (3 mg/kg/day) for 4 weeks on mucosal thickness of small intestine. Data are presented as mean ± SD, using Unpaired *T*-test, *n* = 6. ^a^ significant difference in comparison to adult control; ^a^ (*p* < 0.05), ^b^ significant difference in comparison to juvenile control; ^b^ (*p* < 0.05).

Examination of Mallory’s trichrome stained juvenile sections of the adult control group showed a layer of regularly arranged collagen fibers between the mucosa and the musculosa with few thin fibers extending longitudinally into the cores of the villi. The area % of collagen fibers in the submucosa and lamina propria measured 5.27 ± 0.617 (Figure 9). Examination of Mallory’s trichrome stained jejunal sections of the adult model group showed increased amount of discontinuous and irregularly arranged collagen fibers between the mucosa and the musculosa. Most of the villi had distorted collagen fibers in their cores. The area % of collagen fibers in the submucosa and lamina propria measured 19.54 ± 2.11. The findings demonstrate that the administration of indomethacin induced histological changes similar to those observed in the juvenile group. Notably, the effects of indomethacin were more pronounced in the juvenile group.

##### 3.3.2.2. Assessment of liver tissue

Further, we conducted Mallory’s Trichrome to assess the extent of collagen fiber deposition within the hepatocytes, central vein, and portal tracts in both the juvenile and adult groups. This staining technique provided valuable insights into the amount of collagen fiber accumulation in these specific areas, allowing for a better understanding of the fibrotic changes in the liver.

###### 3.3.2.2.1. Juvenile group

**Figure 10:**
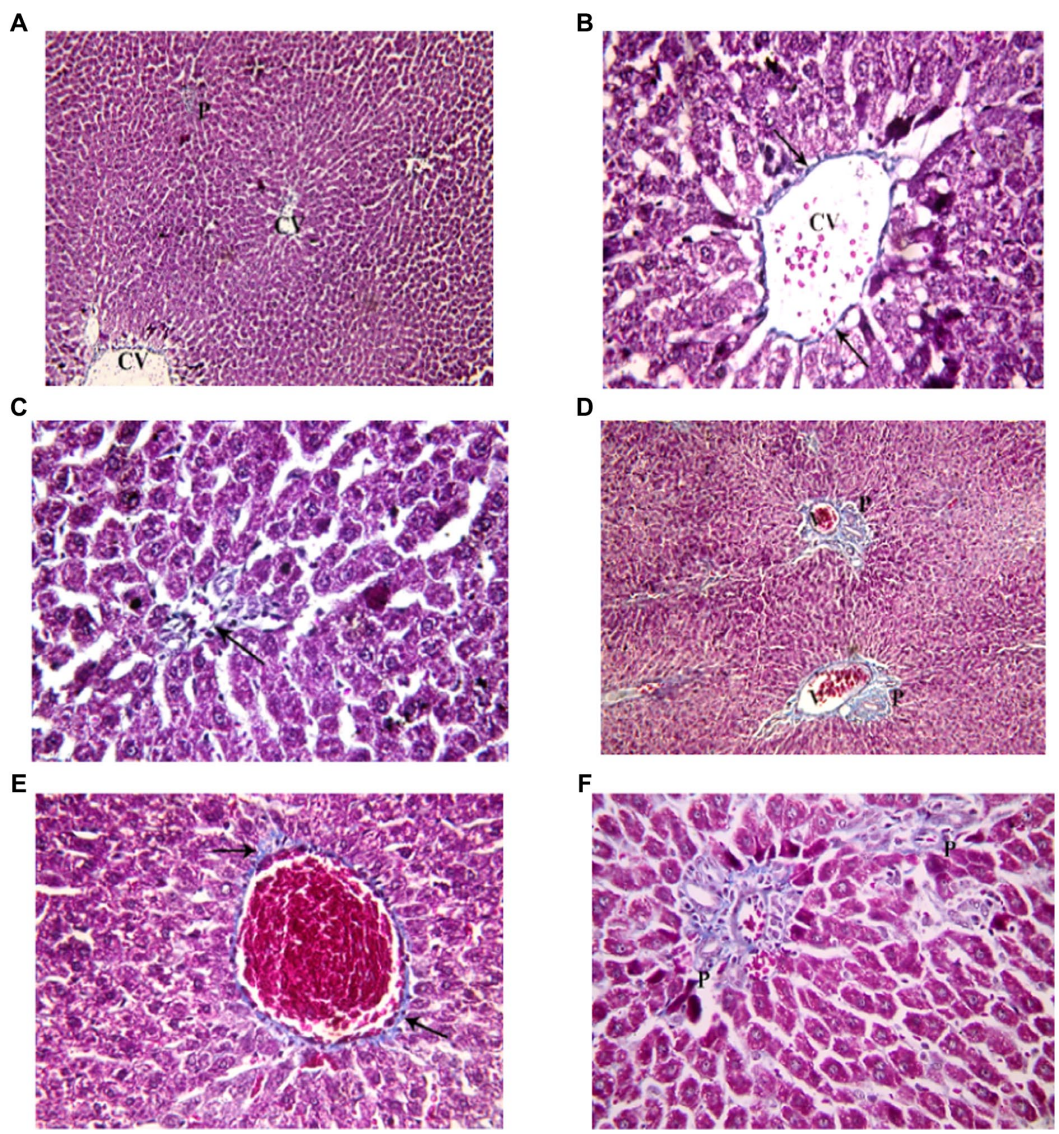
Showing liver sections in juvenile groups. **(A–C)** Control, showing few, thin collagen fibers around central vein (CV) and in portal area (P). **(D–F)** Indomethacin (Model) group. **(D)** Enlarged portal areas (P) with increased amount of collagen fibers. **(E)** Increased amount of collagen fibers (↑) around a markedly congested central vein. **(F)** Collagen fibers extending between adjacent portal areas (P). *Mallory’s trichrome stain: ***(A,D)*** × 100; ***(B,C,E,F)*** × 400.*

Examination of Mallory’s trichrome stained liver sections of the juvenile control group showed few, thin collagen fibers in portal areas and around central veins. The area % of collagen fibers in the liver measured 6.46 ± 1.03 (Figure 10). Examination of Mallory’s trichrome stained liver sections of the juvenile model group showed enlarged portal areas with moderately increased amount of collagen fibers that appeared extending between adjacent portal areas. There was also increased collagen fibers around central veins. The area % of collagen fibers in the liver measured 9.93 ± 1.24. The histological findings presented demonstrate that the administration of indomethacin leads to an increase in the deposition of collagen fibers in the liver parenchymal tissue compared to the control group. This increase may be attributed to the translocation of intestinal bacteria into the circulation, which triggers immune system activation. Subsequently, this activation leads to the infiltration of inflammatory cells, ultimately inducing the activation of hepatic stellate cells and resulting in collagen deposition.

###### 3.3.2.2.2. Adult group

**Figure 11:**
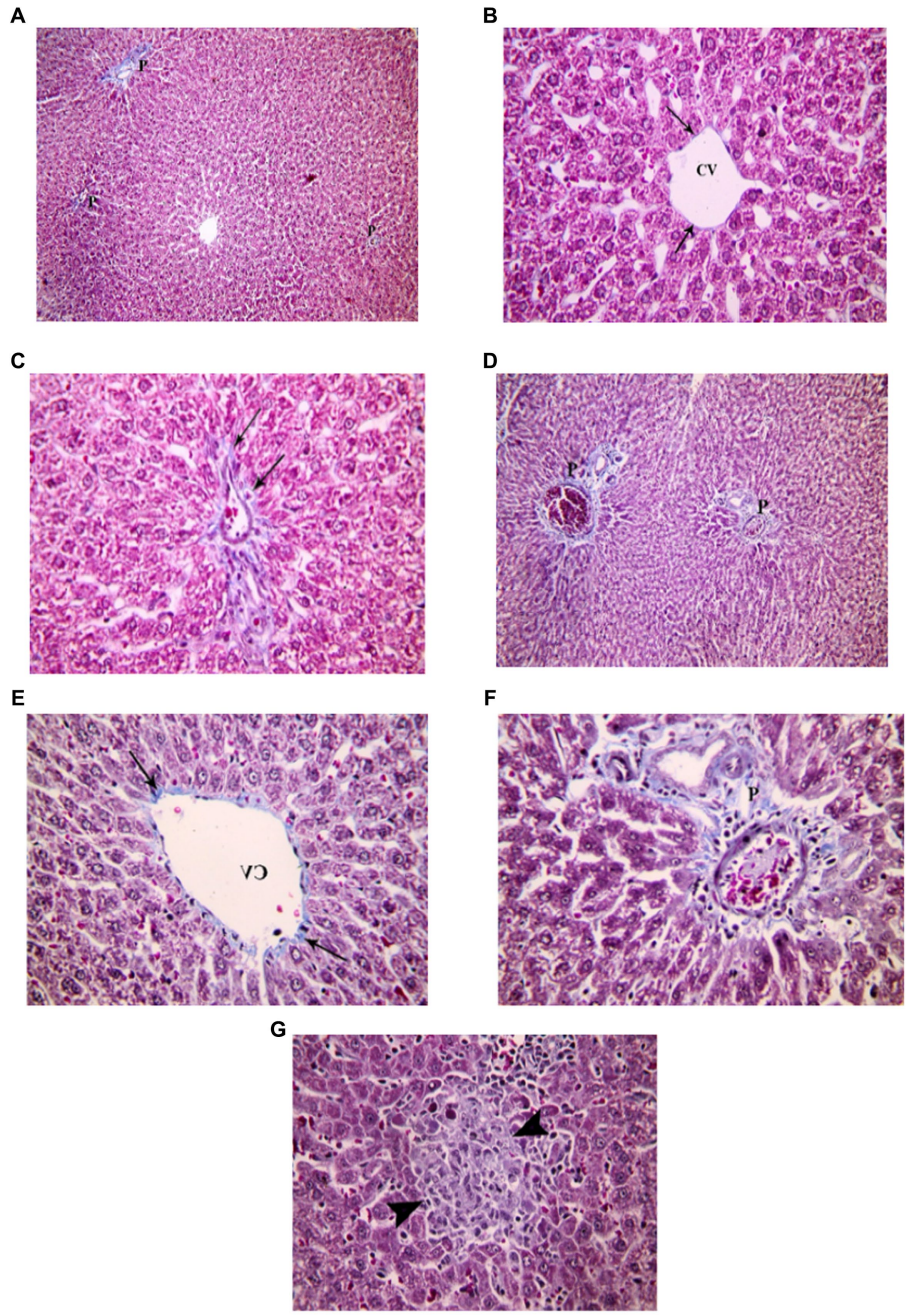
Showing liver sections in adult groups. **(A–C)** Control. **(A)** Collagen fibers in portal areas (P). **(B)** showing thin collagen fibers (↑) around a central vein (CV). **(C)** Section showing few, thin collagen fibers (↑) in a portal area. **(D–G)** Indomethacin group. **(D)** Enlarged portal areas (P), with increased amount of collagen fibers. **(E)** Increased amount of collagen fibers (↑) around a central vein (CV). **(F)** Enlarged portal area (P), with increased amount of collagen fibers. **(G)** Focal necrosis (▴) seen within a hepatic lobule. *Mallory’s trichrome stain: ***(A,D)*** × 100; ***(B,C,E,F,G)*** × 400.*

Examination of Mallory’s trichrome stained liver sections of the adult control group showed few, thin collagen fibers in portal areas and around central veins. The area % of collagen fibers in the liver measured 5.27 ± 0.852 (Figure 11). Assessment of Mallory’s trichrome stained liver sections of the adult model group showed enlarged portal areas with moderately increased amount of collagen fibers that appeared extending between adjacent portal areas. There was also increased collagen fibers around central veins. Necrotic areas with central fibrosis were also seen. The area % of collagen fibers in the liver measured 7.72 ± 1.44. Based on the preceding histological findings, it can be inferred that indomethacin elicited similar histological effects as observed in the juvenile group. Notably, the impact was more prominent in the juvenile group, highlighting a greater susceptibility to indomethacin-induced changes compared to adult group.

#### 3.3.3. Transmission electron microscopic (TEM) analysis

##### 3.3.3.1. Assessment of intestinal mucosa tissue

To obtain a comprehensive understanding of the ultrastructural changes occurring in various cell types of the intestinal wall, such as enterocytes, goblet cells, microvilli, junctional complexes, and inflammatory cells, transmission electron microscopy (TEM) was utilized to examine the jejunum. This technique allowed for a detailed analysis of the cellular structures and provided insights into the alterations taking place at the subcellular level.

###### 3.3.3.1.1. Juvenile group

**Figure 12:**
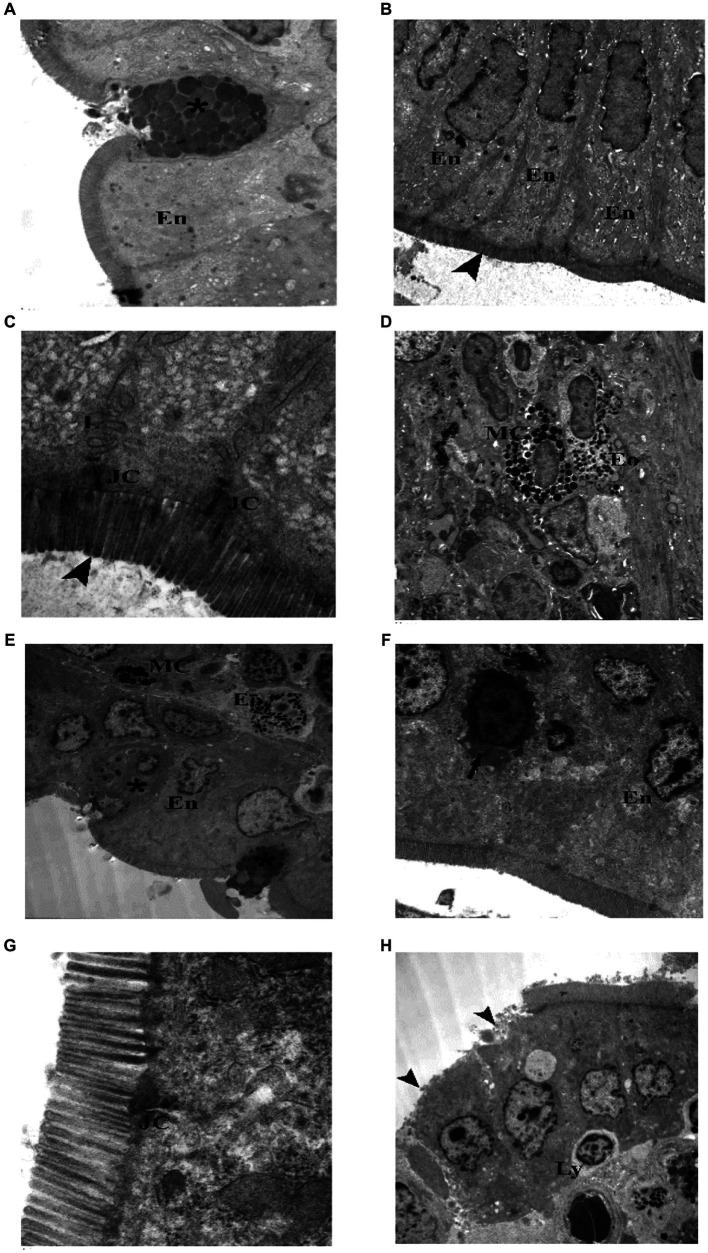
Electron photomicrographs of the intestine of the juvenile groups. **(A–D)** Control. **(A)** Showing normal enterocytes (En) and goblet cell (*) with well defined, homogenous granules. **(B)** Showing enterocytes (En) with oval, basal, euchromatic nuclei and intact microvilli (▴). **(C)** Showing junctional complex (JC), lateral membrane interdigitations (L) and microvilli (▴). **(D)** Showing a mast cell (MC) with electron dens granules and an eosinophil (Eo) in the subepithelial layer. **(E–H)** Indomethacin (Model) group. **(E)** Enterocytes (En) have irregular nuclei, goblet cells (*) have ill defined, heterogenous secretory granules, Mast cell (M) (degranulated) and an eosinophil (Eo). **(F)** Enterocytes (En) with irregular nuclei, one enterocyte has shrunken electron dens cytoplasm and nucleus (↑). **(G)** Junctional complex (JC) appear widely separated. **(H)** Enterocytes with loss of their microvilli (▴), intraepithelial lymphocyte (Ly). *TEM: ***(D,E,H)*** × 1,000; ***(A,B,F)*** × 1,500; ***(C,G)*** × 7,500*.

TEM examination of the jejunum of the juvenile control group showed enterocytes with oval, basal, euchromatic nuclei and intact microvilli, goblet cell appeared full of well defined, homogenous (nearly of same density and size), secretory granules. Junctional complex was seen with its components (tight junction, adhering junction and desmosomes). Lateral membrane interdigitations were clear. Microvilli were seen intact, regular with equal lengths and widths. Occasionally, mast cells and eosinophils were seen in the subepithelial layer (Figure 3). TEM examination of the jejunum of the juvenile model group showed most enterocytes with irregular nuclei, few enterocytes had shrunken electron dens cytoplasm and nuclei. Goblet cells had ill defined, heterogenous (with variable densities and size) secretory granules. Mast cell (degranulated) and eosinophils were seen in the subepithelial layer. Junctional complex appeared widely separated. Some areas showed enterocytes with loss of their microvilli. Intraepithelial lymphocytes were present (Figure 12). The findings suggest that the administration of indomethacin leads to a reduction in the tight junctions and adherence between the microvilli, causing distortion of the enterocytes. Additionally, there is infiltration of mast cells and eosinophils. This effect highlights the ability of indomethacin to disrupt the intestinal barrier, leading to dysbiosis.

###### 3.3.3.1.2. Adult group

**Figure 13:**
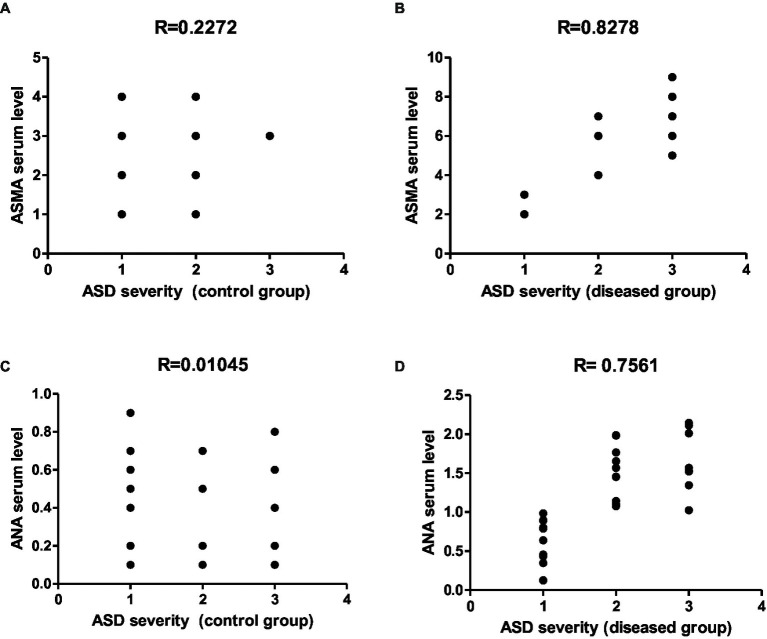
Representative graphs showed the correlation between ASMA and ANA expression and the degree of ASD severity in control **(A,C)** and disease groups **(B,D)**; where 1 = mild social communication & 2 = moderate restricted & 3 = repetitive behaviors.

**Figure 14:**
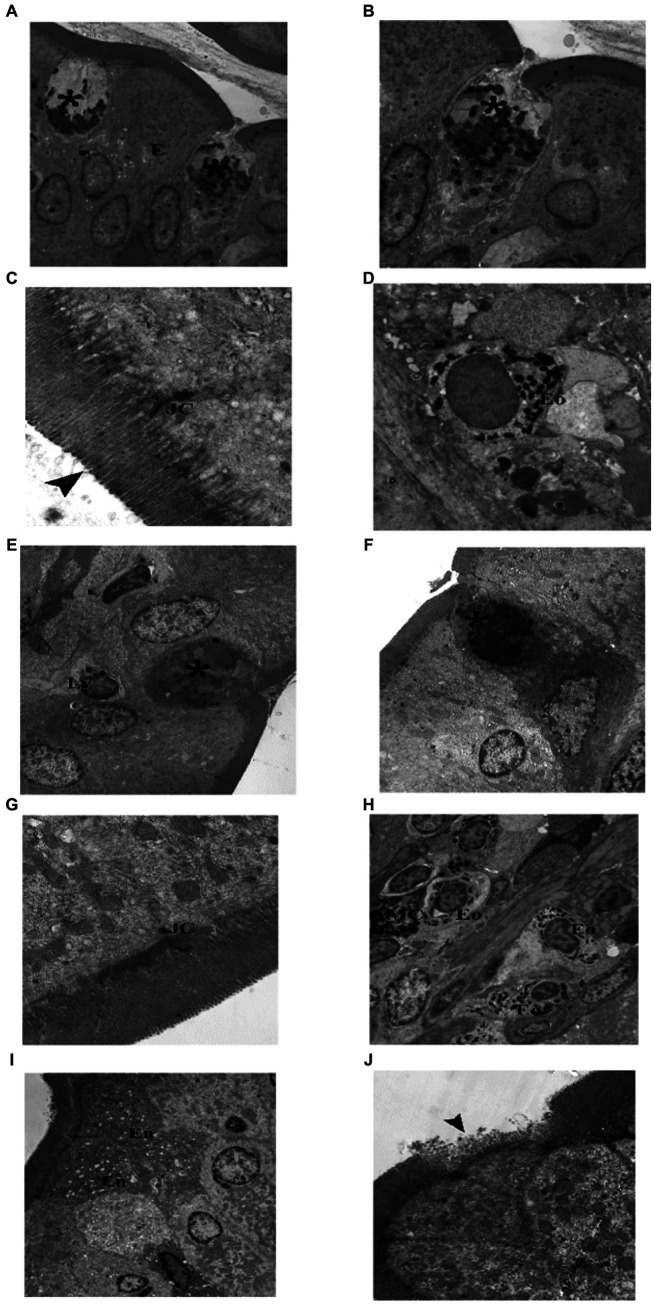
Electron photomicrographs of the intestine of the adult groups. **(A–D)** Control. **(A)** Showing normal enterocytes (E) with oval nuclei, and goblet cells (*). **(B)** A goblet cell (*) with electronwell defined, homogenous dens secretory granules and rER. **(C)** Showing junctional complex (JC), and microvilli (▴). **(D)** An eosinophil (Eo) in the subepithelial layer. **(E–J)** Indomethacin (Model) group. **(E)** A goblet cell (*) with few ill defined, heterogenous secretory granules, and intraepithelial lymphocyte (Ly). **(F)** A goblet cell (*) with ill defined, heterogenous secretory granules and electron dens cytoplasm. **(G)** Showing abnormal (short and destructed) junctional complex (JC). **(H)** Inflammatory infiltrate in subepithelial layer mainly eosinophils (Eo) and mast cells (MC). **(I)** Showing enterocytes (En) with electron dens, vacuolated cytoplasm. **(J)** ENTEROCYTES with loss of their microvilli (▴). *TEM: ***(A,E,H,I)**** *× 1,000*; ***(B,F)*** *× 1,500*; ***(D,J)*** *× 2000*; ***(G)*** *× 4,000*; ***(C)*** *× 6,000*.

TEM examination of the jejunum of the adult control group showed normal enterocytes with oval nuclei, and goblet cells with electron dens secretory granules and rER. Components of junctional complex were clear, and microvilli were regular and intact. Few eosinophils were found in the subepithelial layer (Figure 13). TEM examination of the jejunum of the adult model group showed some enterocytes with electron dens, vacuolated cytoplasm, goblet cells had electron lucent secretory granules and condensed electron dens cytoplasm. Junctional complex appeared abnormal (short and destructed) as compared to the control group. Inflammatory infiltrate seen in subepithelial layer contained many eosinophils and mast cells. In some areas, enterocytes appeared with loss of their microvilli (Figure 14). Based on the previous histological findings, it can be concluded that indomethacin administration resulted in histological changes similar to those observed in the juvenile group. Notably, the effects of indomethacin were more pronounced in the juvenile group.

##### 3.3.3.2. Assessment of liver tissue

To examine the effect of indomethacin administration on the ultrastructure of liver tissue, TEM analysis was applied on liver sections.

###### 3.3.3.2.1. Juvenile group

**Figure 15:**
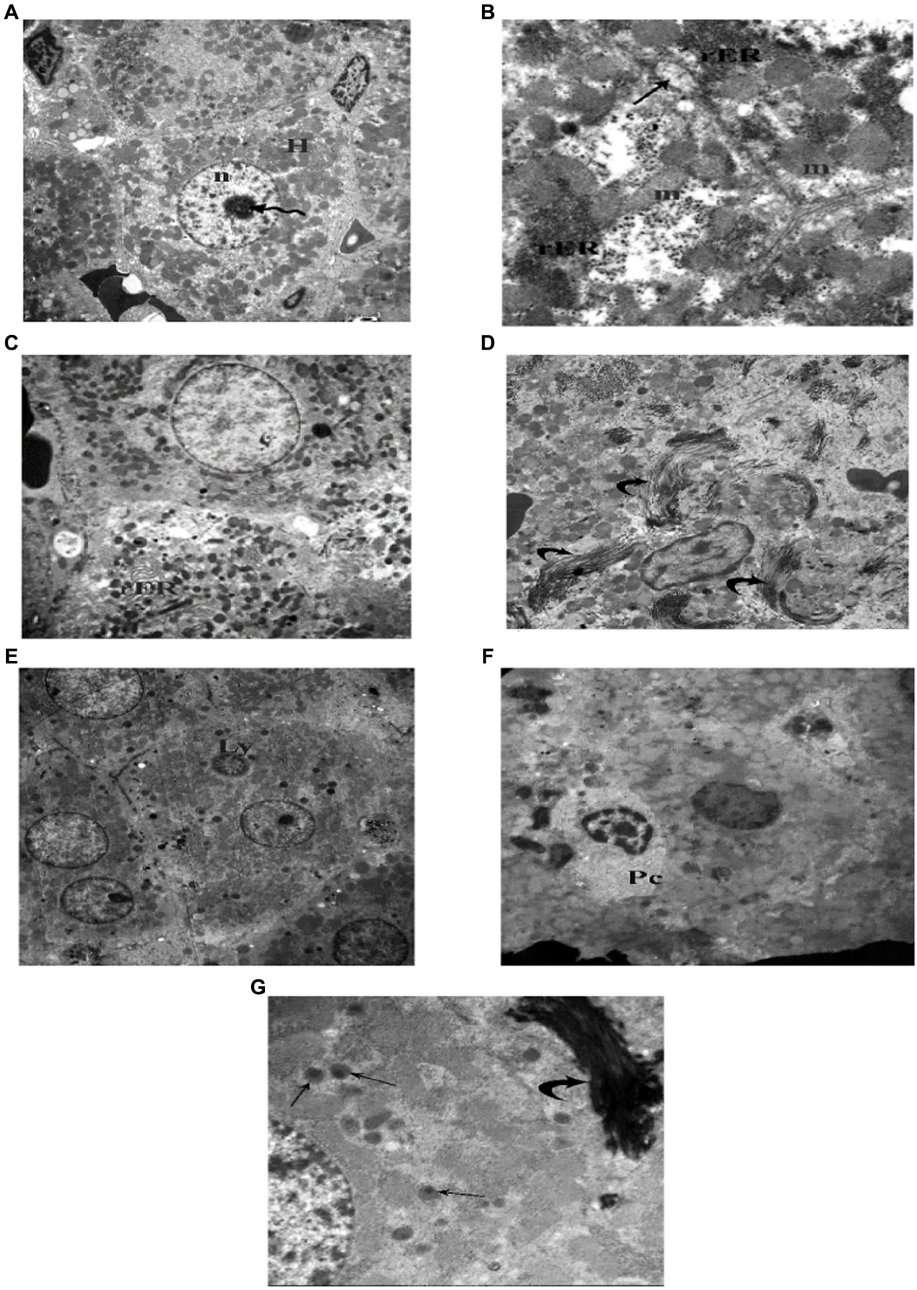
Electron photomicrographs of the liver of the juvenile groups. **(A,B)** Control. **(A)** Showing polygonal hepatocyte (H) with euchromatic central rounded nucleus (n) and prominent nucleolus (waved arrow). **(B)** Showing mitochondria (m), rough endoplasmic reticulum (rER) and a bile canaliculus (↑). **(C–G)** Indomethacin (Model) group. **(C)** Showing rER with dilated, distorted cisternae. **(D)** Showing collagen fibers (curved arrow) deposited in between hepatocytes. **(E)** Showing a lymphocyte (Ly) inside a hepatocyte (Emperipolesis). **(F)** A plasma cell (Pc) in between hepatocytes. **(G)** Many peroxisomes (↑) in a hepatocyte and a bundle of collagen fibers (▴). v*TEM: ***(A,E)*** × 1,000; ***(C,D,F)*** × 1,200; ***(B,G)*** × 3,000*.

TEM examination of the liver of the juvenile control group showed hepatocyte polygonal in shape with euchromatic central rounded nuclei and prominent nucleoli. Their cytoplasm contained numerous mitochondria and regular, closely packed cisternae of rough endoplasmic reticulum (rER). Sometimes bile canaliculi were seen between adjacent hepatocytes (Figure 15). TEM examination of the liver of the juvenile model group showed hepatocytes with dilated, distorted cisternae of rER. Occasionally a lymphocyte could be seen inside a hepatocyte (Emperipolesis). Plasma cells appeared in between hepatocytes. Hepatocytes contained many peroxisomes in their cytoplasm. Bundle of collagen fibers were deposited in between hepatocytes (Figure 15). Based on these histological findings, it can be concluded that the administration of indomethacin triggered the activation of the immune system by inducing intestinal bacterial translocation. As a consequence, lymphocytes and plasma cells targeted the liver cells, leading to hepatocyte damage.

#### 3.3.4. Immunohistochemical analysis

**Figure 16:**
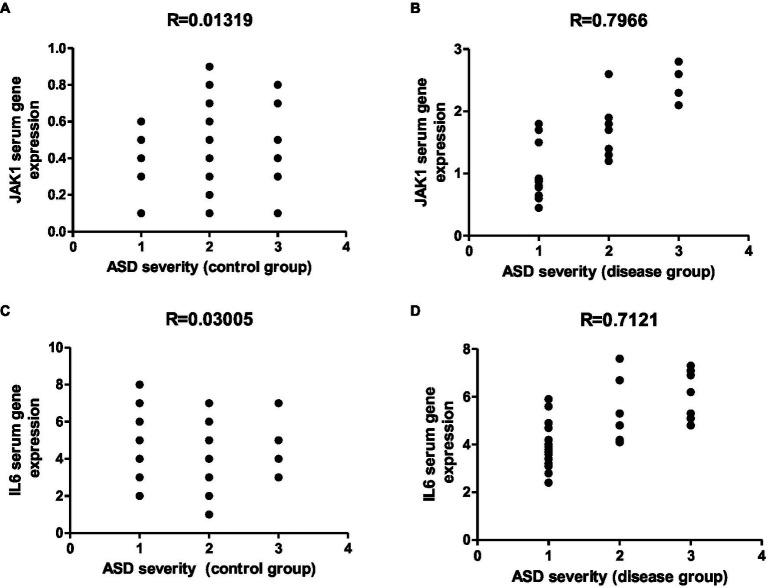
Representative graphs showed the correlation between JAK1 and IL-6 expression and degree of ASD severity in control **(A,C)** and disease groups **(B,D)**; where 1 = mild social communication & 2 = moderate restricted & 3 = repetitive behaviors.

**Figure 17:**
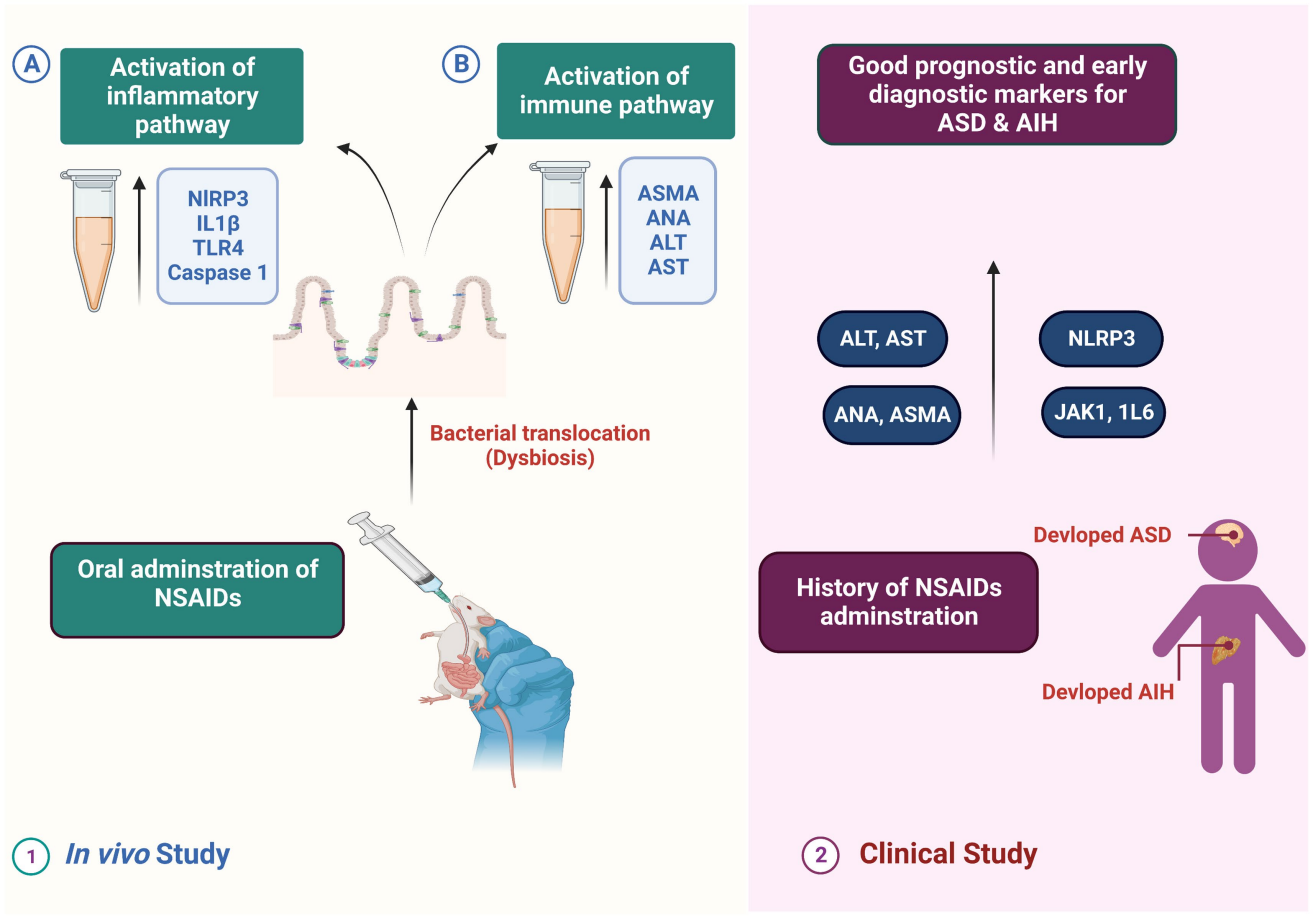
Representative diagram for the proposed mode of action of NSAID-induced dysbiosis on the development of AIH and severity of ASA.

**Figure 18:**
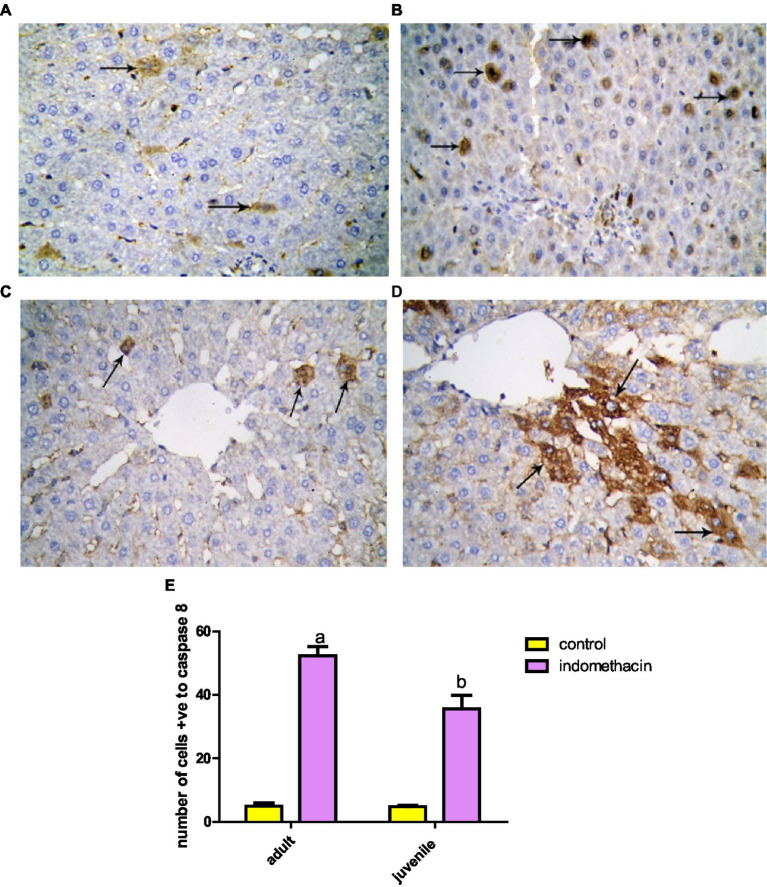
Showing liver sections with Caspase-8 immunostaining in juvenile **(A,B)** and adult **(C,D)** groups. **(A,C)** Control, showing few hepatocytes with positive expression of Caspase-8 (↑). **(B,D)** Indomethacin group, showing many hepatocytes with positive expression of Caspase-8 (↑). *Caspase-8: ***(A,D)*** × 400*. **(E)** Effect of daily oral administration of indomethacin (3 mg/kg, 4 weeks) on the number of cells positive to caspase 8. Data are presented as mean ± SD using Unpaired *T*-test. *n* = 6. ^a^ significant difference in comparison to adult control; ^a^ (*p* < 0.05), ^b^ significant difference in comparison to juvenile control; ^b^ (*p* < 0.05).

To examine the effect of indomethacin administration on the activation of caspase 8 expression, detailed immunohistochemical study was conducted. Examination of immunohistochemically stained liver sections for detection of positive expression of Caspase-8 in the juvenile control group showed minimal reaction. The number of hepatocytes with positive expression of Caspase-8 revealed 4.50 ± 0.428 (Figures 16, 17). Assessment of immunohistochemically stained liver sections for detection of positive expression of Caspase-8 in the juvenile model group showed significantly increased reaction. The number of hepatocytes with positive expression of Caspase-8 measured as 38.67 ± 2.31 (Figure 18). These results emphasize the impact of indomethacin-induced dysbiosis on increasing the expression of caspase 8, resulting in the subsequent activation of the NLRP3 inflammasome pathway.

Further, the expression of caspase 8 in liver tissue in adult group was examined by immunohistochemistry study. Investigation of immunohistochemically stained liver sections for detection of positive expression of caspase-8 in the adult control group showed few hepatocytes with positive expression of Caspase-8. They appeared with dark brown reaction in their cytoplasm. The number of hepatocytes with positive expression of Caspase-8 measured as 5.66 ± 0.494 (Figure 17). Assessment of immunohistochemically stained liver sections for detection of positive expression of caspase-8 in the adult-treated group showed significantly increased reaction. The number of hepatocytes with positive expression of caspase-8 assessed as 51.33 ± 3.39 (Figure 18). These histological findings suggest that indomethacin induces similar histological changes as observed in the juvenile group. However, the effects of indomethacin were notably more pronounced in the juvenile group.

**Figure 19:**
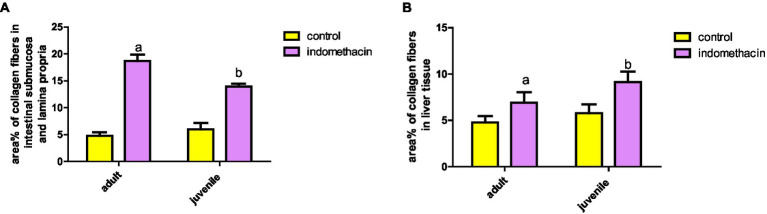
Effect of daily oral administration of indomethacin (3 mg/kg, 4 weeks) on the area% of collagen fibers in intestinal submucosa and lamina propria **(A)**, and in liver tissue **(B)**. Data are presented as mean ± SD using Unpaired *T*-test. n = 6. ^a^ significant difference in comparison to adult control; ^a^ (*p* < 0.05), ^b^ significant difference in comparison to juvenile control; ^b^ (*p* < 0.05).

We conducted further analysis to evaluate the extent of collagen fiber area in the small intestine of both the juvenile and adult groups. The results demonstrated that the untreated groups of both juveniles and adults exhibited a normal collagen fiber area, measuring 4.83 and 5.27%, respectively. In contrast, when indomethacin (3 mg/kg/day) was administered for a duration of 4 weeks, there was a significant (*p* < 0.05) increase in the collagen fiber area (%) within the submucosa and lamina propria of the small intestine. The juvenile treated group showed an area of 14.32%, while the adult treated group exhibited an area of 19.54%, as compared to the control groups (Figure 19). Lastly, we evaluated the area of collagen fibers in the liver tissue of both the juvenile and adult groups. Similar to the results observed in the small intestine, the untreated groups exhibited a normal range of collagen fiber area in both the juvenile (6.46%) and adult (5.27%) groups. However, when indomethacin was administered at a dosage of 3 mg/kg/day for a duration of 4 weeks, there was a significant (*p* < 0.01) increase in the percentage of collagen fiber area in the liver tissue of both the juvenile (9.93%) and adult (7.72%) treated groups, as compared to the control groups (Figure 19). The findings of increased collagen fiber area in the intestinal submucosa and lamina propria in the indomethacin-treated group compared to the control group suggest a potential association between indomethacin administration, intestinal fibrosis, and the development of AIH in the context of ASD.

### 3.4. Clinical studies

NSAIDs drugs are administered to a significant number of infants and young children during their early stages of life. These medications serve as pain relievers, fever reducers, and are also used to promote the closure of patent ductus arteriosis (Ziesenitz et al., 2022). Notable medical records of children with ASD have revealed that a certain proportion of them were administered NSAIDs during their early years, while another percentage was diagnosed with autoimmune liver disease (Hodges et al., 2020). Encouraged by these facts and by our biochemical, histological and immunological findings, we hypothesized that there is a close association between indomethacin administration, intestinal dysbiosis, AIH, and ASD severity, especially in the juvenile group. Accordingly, we conducted an initial clinical study with the objective of investigating the relationship between the markers identified in our *in vivo* experimental research and the level of severity observed in individuals with ASD. Furthermore, our objective was to explore the potential molecular connection between autoimmune liver disease and ASD, ultimately establishing the relationship between dysbiosis, AIH, and the severity of ASD symptoms. The identification of this correlation between the potential markers and the severity of ASD could serve as a valuable tool for early diagnosis and prognosis of the disease, particularly in children who have been exposed to NSAIDs during their early years and are also affected by autoimmune liver disease.

#### 3.4.1. Exploration of the correlation between liver function test and degree of ASD severity

**Figure 20:**
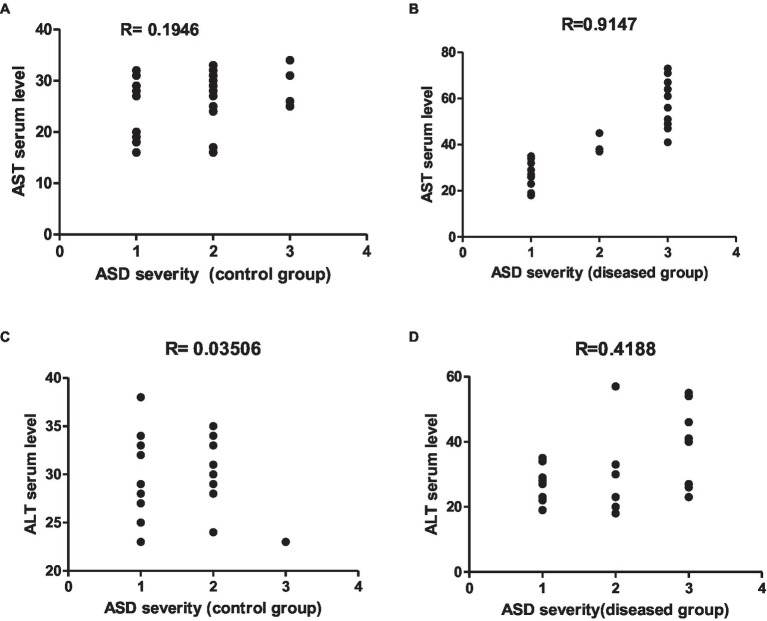
Representative graphs showed the correlation between serum ALT and AST levels and the degree of ASD severity in control **(A,C)** and disease groups **(B,D)**; where 1 = mild social communication & 2 = moderate restricted & 3 = repetitive behaviors.

To investigate the possible association between impaired liver function and the severity of ASD symptoms, we assessed the serum ALT and AST levels in control and disease group and conducted a correlation study. In control group, the results revealed a non-significant correlation between serum ALT and AST levels and ASD severity, (*R* = 0.03506) and (*R* = 0.1946), respectively (Figure 20). On the other hand, there was a significant (*p* < 0.001) positive correlation between serum ALT (*R* = 0.4188) and AST (*R* = 0.9147) levels and the degree of ASD severity in the disease group (Figure 20). The significant positive correlation between serum ALT and AST levels and the degree of ASD severity in the disease group suggests a potential link between compromised liver function and the severity of ASD symptoms.

#### 3.4.2. Exploration of the correlation between autoimmune markers (ASMA and ANA) and the degree of ASD severity

ASMA and ANA are widely recognized markers that exhibit elevated levels in various autoimmune diseases, particularly in cases of autoimmune liver disease (Zeman and Hirschfield, 2010). To affirm the association between autoimmune liver disease and ASD severity, we assessed the expression of ASMA and ANA markers and performed a correlation study analysis toward ASD severity in control and diseased group. As displayed in Figure 13, the results demonstrated a non-significant correlation between ASMA and ANA serum levels and ASD severity (*R* = 0.2272) & (*R* = 0.01045), respectively. Interestingly, in the diseased group, the results revealed a significant (*p* < 0.001) positive correlation between ASMA and ANA expression levels and the degree of ASD severity, (*R* = 0.8278) & (*R* = 0.7561), respectively (Figure 13). ANA and ASMA are peripheral inflammatory markers recognized for their involvement in autoimmune liver diseases, and research has indicated their heightened expression in children who were administered NSAIDs during their early years (Muratori et al., 2023). Moreover, these markers are found to be highly expressed in children with ASD history (Higazi et al., 2021). Considering these facts and our findings, a potential connection may emerge between the disruption of intestinal permeability induced by NSAIDs and the progression of ASD severity in children. Furthermore, our results underscore the potential significance of ASMA and ANA expression as promising prognostic markers for children with ASD and a history of autoimmune liver disease.

#### 3.4.3. Exploration of the correlation between inflammatory markers (NLRP3 & IL18) and degree of ASD severity

**Figure 21:**
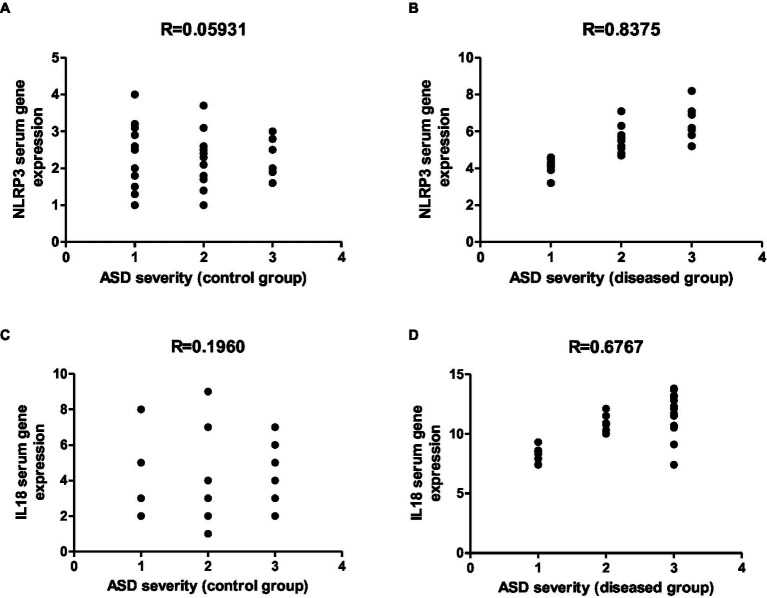
Representative graph showed the correlation between serum NLRP3 and IL18 expression levels and the degree of ASD severity in control **(A,C)** and disease groups **(B,D)**; where 1 = mild social communication & 2 = moderate restricted & 3 = repetitive behaviors.

To affirm the connection between the activation of the NLPR3 inflammasome pathway in children exposed to NSAIDs and the level of severity of ASD, the expression of serum NLRP3 and IL18 genes were examined and a correlation study analysis with ASD severity in the control and diseased groups was performed. In agreement with our hypothesis, the results demonstrated a non-significant correlation between expression levels of NLRP3 and IL-18 genes and the degree of ASD severity in the control group (*R* = 0.05931) and (*R* = 0.1960), respectively. Meanwhile, a significant (*p* < 0.0001) positive correlation between expression levels of NLRP3 and IL-18 genes and the degree of ASD severity (*R* = 0.8375) and (*R* = 0.6767), respectively were noticed in the diseased group (Figure 21). NLRP3 inflammasome plays key role in the modulation of liver inflammation and fibrosis through NLRP3/IL-18 inflammasome pathway. Further, NLRP3 inflammasome pathway is highly related to intestinal dysbiosis and autoimmune reaction progression in the liver tissue (Blevins et al., 2022). In numerous psychiatric disorders, elevated levels of IL-1 and IL-18 serve as indicators for the activation of the NLRP3 inflammasome. The NLRP3 inflammasome, being a valuable diagnostic biomarker, has been previously characterized in various mental health conditions, encompassing depression, Alzheimer’s disease, anxiety disorders, cognitive impairments, post-traumatic stress disorder, and autism spectrum disorders (Çelik et al., 2022). In children with ASD, an increase in the NLRP3 inflammasome and another inflammasome complex, absent in melanoma 2, and accordingly the production of IL-1β and IL-18 inflammatory cytokines were observed. Central and peripheral IL-1β reduces neurogenesis and increases anxiety, stress, and abnormal social interaction (Masi et al., 2017). Our presented findings revealed that the elevated levels of NLRP3/IL-18 inflammatory markers are also associated with the severity of ASD in children who previously received NSAIDs. Taken together, these results affirm the correlation between NSAIDs-induced dysbiosis, AIH, and ASD severity, and further, suggest NLPR3 and IL18 genes as appropriate prognostic markers for early diagnosis of ASD children with AIH history.

#### 3.4.4. Exploration of the correlation between neuronal inflammatory markers (JAK1 and IL-6 expression) and degree of ASD severity

Finally, to affirm the role of AIH and ASD in children exposed to NSAIDs in their early life, we have assessed the expression of JAK1 and IL6 genes expression and a correlation analysis study with ASD severity has been conducted in both control and diseased groups. As shown in Figure 16, a non-significant correlation between expression levels of JAK1 (R = 0.01319) and IL-6 (R = 0.03005) genes and the degree of ASD severity was observed in the control group. Conversely, there was a significant (*p* < 0.0001) positive correlation between expression levels of JAK1 and IL-6 genes and the degree of ASD severity, (*R* = 0.7966) and (*R* = 0.7121), respectively, in the diseased group (Figure 16). Previous reports revealed that JAK1 expression is highly elevated in children with liver disease and that JAK1-signaling pathway is significantly activated in children with ASD, suggesting the association of neuroinflammation (Jiang et al., 2022). Further, the expression of JAK1 is strongly linked to increased levels of IL-6, which has been demonstrated to disrupt the adhesion and movement of neuronal cells. This disruption ultimately results in an imbalance between excitatory and inhibitory circuits in individuals with ASD (Shen et al., 2021). Taken together, our findings suggest that children who develop autoimmune hepatitis as a result of NSAIDs-induced dysbiosis may be at risk of experiencing autism spectrum disorder during their lifetime.

## 4. Discussion

Autism spectrum disorder is influenced by a diversity of genetic, environmental, and immunological factors (Matelski and Van de Water, 2016). Moreover, pharmaceutical drugs, toxicants, metabolic and nutritional factors, and immunologic risk factors have been identified as increasing autism risk (Delaye et al., 2018). However, the clinical diagnosis is founded primarily on the development of complex behavioral disorders by the age of 12–18 months (Wan et al., 2013). Therefore, the proposed therapies that need more investigation include special diets, probiotics, immune modulation, oxytocin, and personalized pharmacogenomic targets (Bharathi et al., 2019; Quaranta et al., 2019). Given the need for evidence to establish the correlation between ASD and autoimmune mechanisms, the aim was to identify a specific immunological signature for ASD to have a better understanding of the link between ASD, dysbiosis, and AIH.

In our experimental study indomethacin produced AIH and this was confirmed by the histopathological examination which revealed the presence of salient features of AIH. Inflammatory cell infiltration including lymphocytes and plasma cells was found in indomethacin-treated rats both juvenile and adult animals. Multinucleated giant cells, rosettes, and intact lymphocytes were found in hepatic tissue. Aggregation of the inflammatory cells around the central vain and extending beyond the portal area – interphase hepatitis. Moreover, intact lymphocytes were noticed inside the hepatocytes giving rise to the characteristic feature of emperipolesis. Furthermore, collagen fibers were abundant in hepatic tissue detected by Mallory’s trichrome in both juvenile and adult animals. In addition, Caspase-8 was abundantly expressed in both juvenile and adult indomethacin-treated rats with significantly increased reaction compared to the control group. These features are typical of AIH as recommended by the American Association for the Study of Liver diseases (AASLD) (Manns et al., 2010) and the European Association for the Study of the Liver (EASL) (EASL Clinical Practice Guidelines, 2015) guidelines. According to the guidelines, it was stated that the presence of hepatic inflammation irrespective of the degree, interface hepatitis, and plasma cells are the hallmark of the diagnosis of AIH (Covelli et al., 2021). Emperipolesis and hepatic rosette formation are mandatory in the diagnosis of the typical form of AIH as well (Hennes et al., 2008). Consequently, the current study represents a typical model of AIH. Besides, AIH is characterized by the upregulation of apoptosis mechanisms, either intrinsic or extrinsic pathways. Caspase-8 mediates the intrinsic mitochondrial pathway as well as the extrinsic legend-mediated pathway (Czaja, 2014). Therefore, Caspase-8 is considered a therapeutic target in AIH (Dempke et al., 2017; Hendawy, 2017). The detection of Caspase-8 in the indomethacin-treated rats is a strong prediction of AIH in our model (Figure 17).

In the present study, gram-negative bacteria were dominant in blood culture after the administration of indomethacin to both juvenile and adult Wistar rats. The predominant organisms were E-coli, Enterococcus spp., and Klebsiella spp. Non-steroidal anti-inflammatory drugs (NSAID) were established to cause gastrointestinal injury including stomach, duodenum, and distal small intestine -drug-induced enteropathy. Consequently, NSAID induces dysbiosis with the predominance of gram-negative bacteria over gram-positive bacteria (Rogers and Aronoff, 2016; Maseda et al., 2019). However, the dynamics of dysbiosis in the small intestine remain to be elucidated (Zhang et al., 2022). Therefore, the current study is in accordance with the current literature emphasizing the significant role of indomethacin, an NSAID, in intestinal epithelial injury and dysbiosis.

In the present study, there was increased thickness of the mucosa and the width of the villi. The indomethacin-received groups showed swollen and distorted villi with sloughed tips. Most of the enterocytes appeared cubical with rounded nuclei. The core of the villi was markedly vacuolated (edematous). The goblet cells showed ill-defined secretory granules with diverse densities and sizes. Most of the villi were with unapparent collagen fibers. However, collagen fibers were abundant in the submucosa. Submucosa collagen fibers were discontinuous and irregular. MNC infiltration was noticed as well.

After a single subcutaneous injection of indomethacin (10 mg/kg), Lang et al. (1988) detected two brief terminal ileal structures that were characterized histologically by submucosal fibrosis and mucosal villous distortion. The same findings were found by Nakajima et al. (2012), and they suggested that edema and neutrophil infiltration could be to blame. In addition, rats who were given indomethacin exhibited symptoms such as granulomatous inflammation, villus atrophy, crypt hyperplasia, and depletion of Paneth cells and goblet cells (Chamoun-Emanuelli et al., 2019). These pathological changes, which were attributed to the administration of indomethacin, were postulated to result in the disruption of the intestinal barrier (analgesic enteropathy) and the leakage of intestinal bacteria and certain toxins, both of which have the potential to reach the liver cells through the portal circulation and induce AIH. Clinical trials provided evidence that lent weight to this perspective (Matsumoto et al., 2006). Matsumoto et al. (2006) reported mild to moderate inflammatory infiltrates in the lamina propria with atrophy along with, apoptotic cells in the epithelia and lymph follicle in the submucosa of the villi that were correlated with NSAID administration.

In this study, hepatic aminotransferase enzymes, AST and ALT, were shown to rise in both indomethacin-treated juvenile and adult rat groups compared to control groups. In addition, a positive correlation was found between the level of hepatic aminotransferase enzymes and ASD severity. The rise of aminotransferase is an indication of liver cell injury (Covelli et al., 2021). Drug-induced AIH, including indomethacin in therapeutic doses, showed a high titer of AST and ALT (Lucas, 2016; Tan et al., 2022). Noteworthy, accumulating evidence from the literature support the impact of NSAIDs, including indomethacin, on small and large intestine leading to dysbiosis in preclinical and clinical studies (Lázár et al., 2021). Furthermore, recent evidence suggests the implication of dysbiosis in the pathogenesis of autism in children (Łukasik et al., 2019; Pan et al., 2022). Moreover, autoimmune inflammation has been implicated in the pathogenesis of autism (Singh, 2009). Taken together, Therefore, the elevation of aminotransferases, noticed in this study, in correlation with ASD suggests the relationship between indomethacin-induced dysbiosis, AIH, and the autistic behavior noticed in children who received NSAIDs (Figure 17).

The current study showed a significant rise of circulating autoantibodies -ANA and ASMA- in indomethacin-treated juvenile and adult rat groups. In addition, these autoantibodies were noticed to be correlated with children with ASD. Circulating autoantibodies are considered the cornerstone in AIH type 1 diagnosis (Wang et al., 2021). Given the similarity of the clinical pattern of drug-induced AIH to idiopathic AIH (Licata et al., 2014), non-steroidal anti-inflammatory drugs (NSAIDs) have been considered culprits in AIH with circulating autoantibodies (Tan et al., 2022). The literature suggested that there is a correlation between ASD and autoantibodies (Sotgiu et al., 2020; Rossignol and Frye, 2021; McLellan et al., 2022) including, but not limited to, ANA, an anti-ribosomal P protein, and anti-endothelial cell antibodies (Zou et al., 2020). Several studies correlated ASD with autoimmune diseases of the mother including rheumatoid arthritis and celiac disease (Atladóttir et al., 2009) and an increase in the serum level of anti-myelin-basic protein autoantibodies (Mostafa and AL-Ayadhi, 2011; Mostafa and Al-Ayadhi, 2013). However, there is lack of comparison control studies to elicit solid evidence (Sotgiu et al., 2020).

On the other hand, the gut microbiota has been emphasized as a key factor in the development of ASD (Hughes et al., 2018). It was concluded that the development and severity of ASD features were correlated with dysbiosis (Iglesias-Vázquez et al., 2020). However, there is a lack of consistency in the reported gut microbiome changes across ASD studies (Ho et al., 2020), Administration of probiotics containing a mixture of *Bifidobacteria, Streptococci* and *Lactobacilli* showed promising results in the treatment of ASD (Fattorusso et al., 2019). Furthermore, microbiota transfer therapy was suggested to restore gut symbiosis, thus improving children with ASD (Kang et al., 2019). Our results, in line with the existing literature, showed a strong association between indomethacin and AIH on the one hand and autoantibodies as a marker of AIH and ASD on the other. Concerning the association between indomethacin and dysbiosis, a possible connection between dysbiosis, AIH, and ASD can be predicted.

The current study showed a significant rise in NLRP3, caspase-1, and IL-18 in indomethacin-treated juvenile and adult groups. There was a positive correlation between both NLRP3 and IL-18 and ASD severity. The NLRP3 inflammasome controlled the innate immune system responses against infection (Pétrilli et al., 2007). Uncontrolled activation of the NLRP3 inflammasome by non-pathogenic host stimuli has been implicated in the development of autoimmune disease (Elliott and Sutterwala, 2015). Caspase-1 mediates the NLRP3 cascade through the activation of inactive pro-IL-18 (Zannetti et al., 2016) leading to the initiation of the innate immune system (Elliott and Sutterwala, 2015). Over-expression of NLRP3 inflammasome and cleaved levels of caspase-1 were reported in Concanavalin A (ConA)-induced hepatitis animal model (Guan et al., 2022). Conversely, the mitigation of hepatocellular damage in NLRP3^−/−^ mice compared to wild-type animals suggested the key role of NLRP3 in the pathogenesis of AIH (Luan et al., 2018). Moreover, caspase-1 activation through NLRP3 mediates interleukin activation including IL-1β and IL-18 leading to hepatic cell damage (Kamo et al., 2013). However, it was speculated that IL-18 rather than IL-1β was abundant in the fatal AIH model with amelioration of the AIH progression following *in-vivo* demonstration of anti-IL-18R (Ikeda et al., 2014). The key role of IL-18 in AIH was further supported by the mitigation of hepatic inflammation through the implementation of caspase-1 inhibitors, IL-18 monoclonal antibodies, and soluble IL-18 inhibitors (Figure 17) (Ikeda et al., 2014).

On the other hand, IL-18 has been implicated in dysbiosis. Accumulating evidence from preclinical studies proved that NLRP3 and IL-18 are implicated in the dysbiosis of gut microbiota with consequent development of liver cell inflammation and hepatocyte damage (Dinarello, 2007; Henao-Mejia et al., 2012). Therefore, the significant increase in the expression of hepatic NLRP3 supports its critical role in the initiation and progression of inflammation through NLRP3/caspase-1/IL-18 inflammasome and the correlation with intestinal dysbiosis. The changes of NLRP3/caspase-1/IL-18 were notable in children with ASD who previously received NSAIDs. Given the implication of indomethacin in dysbiosis and AIH, the connection between the degree of autistic behavior and the elevated level of the inflammasomes biomarkers suggests the close correlation between dysbiosis, AIH, and ASD (Figure 17).

There is accumulating evidence of impaired immunity occurring in individuals with ASD both immediately after birth and through the course of the disease progression (Hughes et al., 2018). Neuroinflammation involving microglia, increased inflammatory cytokine and chemokine production in post-mortem brain tissue, systemic alterations of immune proteins, and reduced immunoglobulin (Ig) antibody production are some of the pieces of evidence that point to the possibility of an immune dysfunction being involved in autism (Onore et al., 2012).

JAK/STAT molecules are essential for cytokine signaling allowing a better understanding of the role of cytokine activation in disease processes (Linossi et al., 2018). JAK is activated by the binding of IL-6 to its receptor IL-6R on the surface of the cell, thus initiating the downstream process (Murakami et al., 2019). Recent studies found that there is a positive correlation between JAK1 and IL-6 expression and ASD severity. JAK1 is a tyrosine kinase belonging to the Janus kinase/ signal transducers and activators of transcription (JAK/STAT) which mediates the downstream pathway of different interleukins such as IL-6, IL-2, and IL-10 (O’Shea et al., 2015). The mutation of JAK1 was concluded to be associated with an autoinflammatory skin disease associated with liver dysfunction and ASD (Takeichi et al., 2022). Furthermore, Ahmed and colleagues found increased mRNA and protein expression for JAK1, pJAK1, STAT5, and pSTAT5 in ASD compared to control suggesting the presence of a correlation between ASD and the upregulation of JAK–STAT signaling (Ahmad et al., 2017).

IL-6 plays a substantial role in promoting anti- and pro- inflammatory outcomes through the activation of IL-6 receptors and the engagement of JAK1, 2, or 3 targets along with a downstream transcription of STAT1 and 3 families (Murakami et al., 2019). Consequently, there is a growing body of evidence associating IL-6, a pro-inflammatory cytokine, with the neuroinflammatory process in 22q11.2 deletion syndrome (Mekori-Domachevsky et al., 2017). Furthermore, social impairment was found to be correlated with the IL-6/IL-10 ratio (Ross et al., 2013). Moreover, it was concluded that a high level of Il-6 impacts the cerebellar granule cells’ histopathology and hence the functionality (Wei et al., 2011). However, strong evidence was lacking to establish the causation between IL-6 and JAK1 abnormalities with autism (Takeichi et al., 2022). Although promising and mechanistically relevant, these results should be extrapolated to autism research with great caution, as a pathophysiological contribution of immune and microglia deficits to ASD has yet to be unambiguously demonstrated (Estes and McAllister, 2015). Therefore, this study provided evidence of the correlation between immune mechanisms with the development of ASD. The findings of this study pointed to the existence of a correlation between the levels of expression of the genes JAK1 and IL-6 and the severity of autism spectrum disorder (ASD). Therefore, further studies are required to establish the link between autoimmune products and ASD (Figure 17).

## 5. Conclusion

In conclusion, our research provides compelling evidence for the unique relationship between indomethacin-induced dysbiosis, autoimmune hepatitis (AIH), and the development of autism spectrum disorder (ASD). Dysbiosis emerged as a critical factor in the development of AIH, subsequently leading to the onset of ASD. The interconnectedness among these components, as supported by our findings, strengthens the hypothesis that the development of ASD may be linked to dysbiosis-induced autoimmune inflammatory processes. We have demonstrated that dysbiosis plays a pivotal role in the development of AIH, which subsequently contributes to the emergence of ASD. Our study revealed that intestinal dysbiosis, as evidenced by elevated serum levels of NLRP3 inflammasomes, TLR4, IL18, caspase1, along with decreased mucosal thickness and increased width between intestinal villi, is closely associated with AIH. Similarly, markers of AIH, such as elevated liver enzymes, circulating autoantibodies, JAK1, IL6, lymphocytes, and plasma cell infiltrates, were identified. Importantly, these markers exhibited a positive correlation with the severity of ASD symptoms, indicating a potential link between dysbiosis-induced autoimmune inflammatory processes and the development of ASD. The findings of our research have significant implications for early diagnosis and prognosis of ASD, particularly in children who have been exposed to non-steroidal anti-inflammatory drugs (NSAIDs) or who have AIH. The possibility of measuring the aforementioned parameters could potentially enable early identification of individuals at risk for developing ASD. Furthermore, these markers may serve as prognostic indicators, helping clinicians to anticipate the progression and severity of ASD in affected individuals. Building upon our findings, we recommend conducting a clinical trial to explore the role of probiotics in children with ASD and its impact on the measured inflammatory and autoimmune markers. Probiotics have shown promise in modulating gut microbiota and addressing dysbiosis-related conditions, and their potential benefits in the context of ASD warrant further investigation. Additionally, our study raises concerns regarding the use of NSAIDs in children. The potential association between NSAIDs, dysbiosis, AIH, and the subsequent development of ASD calls for caution in the administration of these medications to pediatric patients. This finding highlights the importance of raising awareness about the potential hazards associated with NSAID use in children and emphasizes the need for further research and regulatory considerations in this area. Overall, our study sheds light on the intricate relationship between dysbiosis, AIH, and ASD, providing a foundation for further research in this field. The identification of specific biomarkers associated with these conditions opens up new avenues for understanding their pathogenesis and developing targeted interventions. Ultimately, our findings contribute to a deeper understanding of the complex mechanisms underlying ASD and offer hope for improved diagnostic and therapeutic strategies in the future.

## Data availability statement

The original contributions presented in the study are included in the article/supplementary material, further inquiries can be directed to the corresponding authors.

## Ethics statement

The studies involving humans were conducted in accordance with the Declaration of Helsinki and approved by the Institutional Review Board (or Ethics Committee) of Faculty of Medicine, Ain Shams University (FMASUR238/2023). The studies were conducted in accordance with the local legislation and institutional requirements. Written informed consent for participation in this study was provided by the participants’ legal guardians/next of kin. The animal study protocol was approved by the Institutional Review Board (or Ethics Committee) of Faculty of Medicine, Ain Shams University (FMASUR213/2023). The study was conducted in accordance with the local legislation and institutional requirements.

## Author contributions

DM: Conceptualization, Investigation, Methodology, Project administration, Resources, Validation, Writing – original draft, Writing – review & editing. HHA: Data curation, Formal analysis, Methodology, Software, Validation, Visualization, Writing – original draft. AE: Conceptualization, Investigation, Methodology, Project administration, Supervision, Visualization, Writing – original draft. DE-W: Conceptualization, Data curation, Investigation, Methodology, Project administration, Resources, Supervision, Writing – original draft. OE-K: Conceptualization, Investigation, Methodology, Project administration, Resources, Supervision, Writing – original draft, Writing – review & editing, Validation. SM: Formal analysis, Investigation, Methodology, Software, Validation, Visualization, Writing – review & editing. YS: Data curation, Formal analysis, Investigation, Methodology, Software, Validation, Writing – review & editing. RA: Data curation, Formal analysis, Funding acquisition, Investigation, Software, Validation, Visualization, Visualization. HAA: Data curation, Formal analysis, Investigation, Methodology, Resources, Software, Validation, Visualization, Writing – review & editing. AA: Data curation, Investigation, Software, Validation, Visualization, Writing – review & editing, Formal analysis, Funding acquisition, Supervision. II: Data curation, Investigation, Software, Validation, Visualization, Writing – review & editing, Methodology, Resources. SA: Data curation, Formal analysis, Investigation, Software, Validation, Visualization, Writing – review & editing, Funding acquisition. MJ: Data curation, Formal analysis, Investigation, Resources, Software, Validation, Visualization, Writing – original draft, Writing – review & editing, Supervision. A-HE: Formal analysis, Investigation, Methodology, Resources, Writing – original draft, Writing – review & editing, Data curation, Software, Validation, Visualization. ES: Conceptualization, Formal analysis, Funding acquisition, Investigation, Methodology, Project administration, Resources, Supervision, Writing – original draft, Writing – review & editing.

## References

[ref1] Abdel-Wahab B. A. Abd El-Kareem F. Alzamami A. Fahmy C. Elesawy B. Mostafa Mahmoud M.. (2022). Novel exopolysaccharide from marine *Bacillus Subtilis* with broad potential biological activities: insights into antioxidant, anti-inflammatory, cytotoxicity, and anti-Alzheimer activity. Meta 12:715. doi: 10.3390/metabo12080715 PMC9413097 36005587

[ref2] Abo Nahas H. H. Darwish A. M. G. Abd EL-kareem H. F. Abo Nahas Y. H. Mansour S. A. Korra Y. H.. (2022). “Trust your gut: the human gut microbiome in health and disease” in Microbiome-gut-brain Axis: Implications on health. eds. Sayyed R. Z. Khan M. (Singapore: Springer), 53–96.

[ref3] Abrahams B. S. Geschwind D. H. (2008). Advances in autism genetics: on the threshold of a new neurobiology. Nat. Rev. Genet. 9, 341–355. doi: 10.1038/nrg2346, PMID: 18414403 PMC2756414

[ref4] Ahmad S. F. Nadeem A. Ansari M. A. Bakheet S. A. Al-Ayadhi L. Y. Attia S. M. (2017). Upregulation of IL-9 and JAK-STAT Signaling pathway in children with autism. Prog. Neuro-Psychopharmacol. Biol. Psychiatry 79, 472–480. doi: 10.1016/j.pnpbp.2017.08.002, PMID: 28802860

[ref5] Atladóttir H. Ó. Pedersen M. G. Thorsen P. Mortensen P. B. Deleuran B. Eaton W. W.. (2009). Association of Family History of autoimmune diseases and autism spectrum disorders. Pediatrics 124, 687–694. doi: 10.1542/peds.2008-2445, PMID: 19581261

[ref6] Baio J. Wiggins L. Christensen D. L. Maenner M. J. Daniels J. Warren Z.. (2018). Prevalence of autism Spectrum disorder among children aged 8 years - autism and developmental disabilities monitoring network, 11 sites, United States, 2014. MMWR Surveill. Summ. 67, 1–23. doi: 10.15585/mmwr.ss6706a1, PMID: 29701730 PMC5919599

[ref7] Banhart S. Saied E. M. Martini A. Koch S. Aeberhard L. Madela K.. (2014). Improved plaque assay identifies a novel anti-chlamydia ceramide derivative with altered intracellular localization. Antimicrob. Agents Chemother. doi: 10.1128/AAC.03457-14, PMID: 25001308 PMC4135853

[ref8] Baxter E. J. Scott L. M. Campbell P. J. East C. Fourouclas N. Swanton S.. (2005). Acquired mutation of the tyrosine kinase JAK2 in human myeloproliferative disorders. Lancet 365, 1054–1061. doi: 10.1016/S0140-6736(05)71142-9, PMID: 15781101

[ref9] Bazin M. Physiological models in microbiology; CRC Press, (2018). Boca Raton, FL

[ref10] Belkaid Y. Hand T. W. (2014). Role of the microbiota in immunity and inflammation. Cells 157, 121–141. doi: 10.1016/j.cell.2014.03.011, PMID: 24679531 PMC4056765

[ref11] Bergot A.-S. Giri R. Thomas R. (2019). The microbiome and rheumatoid arthritis. Best Pract. Res. Clin. Rheumatol. 33:101497. doi: 10.1016/j.berh.2020.101497, PMID: 32199713

[ref12] Bharathi G. Jayaramayya K. Balasubramanian V. Vellingiri B. (2019). The potential role of rhythmic entrainment and music therapy intervention for individuals with autism spectrum disorders. J. Exerc. Rehabil. 15, 180–186. doi: 10.12965/jer.1836578.289, PMID: 31110998 PMC6509464

[ref13] Blevins H. M. Xu Y. Biby S. Zhang S. (2022). The NLRP3 inflammasome pathway: a review of mechanisms and inhibitors for the treatment of inflammatory diseases. Front. Aging Neurosci. 14:879021. doi: 10.3389/fnagi.2022.879021, PMID: 35754962 PMC9226403

[ref14] Bogdanos D. Baum H. Vergani D. Burroughs A. (2014). The Role of *E. coli* infection in the pathogenesis of primary biliary cirrhosis. Dis. Markers 29, 301–311. doi: 10.3233/DMA-2010-0745 PMC3835537 21297249

[ref15] Bruce-Keller A. J. Salbaum J. M. Luo M. Blanchard E. Taylor C. M. Welsh D. A.. (2015). Obese-type gut microbiota induce neurobehavioral changes in the absence of obesity. Biol. Psychiatry 77, 607–615. doi: 10.1016/j.biopsych.2014.07.012, PMID: 25173628 PMC4297748

[ref16] Çelik S. Kayaaltı A. Erbas O. (2022). NLRP3 inflammasome: a new target in psychiatric disorders. CNS Neurosci. Ther. 2, 392–398. doi: 10.5606/jebms.2021.75681

[ref17] Chamoun-Emanuelli A. M. Bryan L. K. Cohen N. D. Tetrault T. L. Szule J. A. Barhoumi R.. (2019). NSAIDs disrupt intestinal homeostasis by suppressing macroautophagy in intestinal epithelial cells. Sci. Rep. 9:14534. doi: 10.1038/s41598-019-51067-2 31601922 PMC6787209

[ref18] Collins S. M. Bercik P. (2009). The relationship between intestinal microbiota and the central nervous system in Normal gastrointestinal function and disease. Gastroenterology 136, 2003–2014. doi: 10.1053/j.gastro.2009.01.075, PMID: 19457424

[ref19] Covelli C. Sacchi D. Sarcognato S. Cazzagon N. Grillo F. Baciorri F.. (2021). Pathology of autoimmune hepatitis. Pathologica 113, 185–193. doi: 10.32074/1591-951X-241, PMID: 34294936 PMC8299324

[ref20] Czaja A. J. (2014). Targeting apoptosis in autoimmune hepatitis. Dig. Dis. Sci. 59, 2890–2904. doi: 10.1007/s10620-014-3284-2, PMID: 25038736

[ref21] Czaja A. J. Freese D. K. (2002). Diagnosis and treatment of autoimmune hepatitis. Hepatology 36, 479–497. doi: 10.1053/jhep.2002.34944 12143059

[ref22] Delaye J.-B. Patin F. Lagrue E. Le Tilly O. Bruno C. Vuillaume M.-L.. (2018). Post hoc analysis of plasma amino acid profiles: towards a specific pattern in autism Spectrum disorder and intellectual disability. Ann. Clin. Biochem. 55, 543–552. doi: 10.1177/0004563218760351, PMID: 29388433

[ref23] Dempke W. C. M. Fenchel K. Uciechowski P. Dale S. P. (2017). Second- and third-generation drugs for Immuno-oncology treatment—the more the better? Eur. J. Cancer 74, 55–72. doi: 10.1016/j.ejca.2017.01.001, PMID: 28335888

[ref24] Dinarello C. A. (2007). Interleukin-18 and the pathogenesis of inflammatory diseases. Semin. Nephrol. 27, 98–114. doi: 10.1016/j.semnephrol.2006.09.013, PMID: 17336692

[ref25] EASL Clinical Practice Guidelines (2015). Autoimmune Hepatitis. J. Hepatol. 63, 971–1004. doi: 10.1016/j.jhep.2015.06.030 26341719

[ref26] El Azab I. H. Saied E. M. Osman A. A. Mehana A. E. Saad H. A. Elkanzi N. A. (2021). Novel N-bridged Pyrazole-1-carbothioamides with potential antiproliferative activity: design, synthesis, in vitro and in silico studies. Future Med. Chem. 13, 1743–1766. doi: 10.4155/fmc-2021-0066, PMID: 34427113

[ref27] Elliott E. I. Sutterwala F. S. (2015). Initiation and perpetuation of NLRP3 inflammasome activation and assembly. Immunol. Rev. 265, 35–52. doi: 10.1111/imr.12286, PMID: 25879282 PMC4400874

[ref28] Elshaer A. M. El-Kharashi O. A. Hamam G. G. Nabih E. S. Magdy Y. M. Abd El Samad A. A. (2019). Involvement of TLR4/CXCL9/PREX-2 pathway in the development of hepatocellular carcinoma (HCC) and the promising role of early Administration of *Lactobacillus Plantarum* in Wistar rats. Tissue Cell 60, 38–47. doi: 10.1016/j.tice.2019.07.010 31582017

[ref29] Estes M. L. McAllister A. K. (2015). Immune mediators in the brain and peripheral tissues in autism Spectrum disorder. Nat. Rev. Neurosci. 16, 469–486. doi: 10.1038/nrn3978, PMID: 26189694 PMC5650494

[ref30] Evans P. O’Reilly D. Flyer J. N. Soll R. Mitra S. (2021). Indomethacin for symptomatic patent ductus arteriosus in preterm infants. Cochrane Database Syst. Rev. 1, CD013133. doi: 10.1002/14651858.CD013133.pub2 PMC8095061 33448032

[ref31] Fattorusso A. Di Genova L. Dell’Isola G. Mencaroni E. Esposito S. (2019). Autism spectrum disorders and the gut microbiota. Nutrients 11:521. doi: 10.3390/nu11030521, PMID: 30823414 PMC6471505

[ref32] Fowlie P. W. Davis P. G. McGuire W. (2010). Prophylactic intravenous indomethacin for preventing mortality and morbidity in preterm infants. Cochrane Database Syst. Rev. CD000174:2010. doi: 10.1002/14651858.CD000174.pub2 PMC7045285 20614421

[ref33] Franzosa E. A. Sirota-Madi A. Avila-Pacheco J. Fornelos N. Haiser H. J. Reinker S.. (2019). Gut microbiome structure and metabolic activity in inflammatory bowel disease. Nat. Microbiol. 4, 293–305. doi: 10.1038/s41564-018-0306-4, PMID: 30531976 PMC6342642

[ref34] Ghonaim M. Al-Ghamdi A. El-Bana H. Bakr A. Ghoneim E. El-Edel R.. (2005). Autoantibodies in chronic liver disease. Egypt. J. Immunol. 12, 101–111. PMID: 17977215

[ref35] Guan Y. Gu Y. Li H. Liang B. Han C. Zhang Y.. (2022). NLRP3 inflammasome activation mechanism and its role in autoimmune liver disease. Acta Biochim. Biophys. Sin. Shanghai 54, 1577–1586. doi: 10.3724/abbs.2022137 36148948 PMC9828325

[ref36] Helmy Y. A. Taha-Abdelaziz K. Hawwas H. A. E.-H. Ghosh S. AlKafaas S. S. Moawad M. M. M.. (2023). Antimicrobial resistance and recent alternatives to antibiotics for the control of bacterial pathogens with an emphasis on foodborne pathogens. Antibiotics 12:274. doi: 10.3390/antibiotics12020274 36830185 PMC9952301

[ref37] Henao-Mejia J. Elinav E. Jin C. Hao L. Mehal W. Z. Strowig T.. (2012). Inflammasome-mediated dysbiosis regulates progression of NAFLD and obesity. Nature 482, 179–185. doi: 10.1038/nature10809, PMID: 22297845 PMC3276682

[ref38] Hendawy N. (2017). Pentoxifylline attenuates cytokine stress and Fas system in syngeneic liver proteins induced experimental autoimmune hepatitis. Biomed. Pharmacother. 92, 316–323. doi: 10.1016/j.biopha.2017.05.084, PMID: 28551553

[ref39] Hennes E. M. Zeniya M. Czaja A. J. Parés A. Dalekos G. N. Krawitt E. L.. (2008). Simplified criteria for the diagnosis of autoimmune hepatitis. Hepatology 48, 169–176. doi: 10.1002/hep.22322, PMID: 18537184

[ref40] Higazi A. M. Kamel H. M. Abdel-Naeem E. A. Abdullah N. M. Mahrous D. M. Osman A. M. (2021). Expression analysis of selected genes involved in tryptophan metabolic pathways in Egyptian children with autism Spectrum disorder and learning disabilities. Sci. Rep. 11:6931. doi: 10.1038/s41598-021-86162-w 33767242 PMC7994393

[ref41] Ho L. K. H. Tong V. J. W. Syn N. Nagarajan N. Tham E. H. Tay S. K.. (2020). Gut microbiota changes in children with autism Spectrum disorder: a systematic review. Gut. Pathog. 12:6. doi: 10.1186/s13099-020-0346-1 32025243 PMC6996179

[ref42] Hodges H. Fealko C. Soares N. (2020). Autism Spectrum disorder: definition, epidemiology, causes, and clinical evaluation. Transl Pediatr 9, S55–S65. doi: 10.21037/tp.2019.09.09, PMID: 32206584 PMC7082249

[ref43] Horobin R. W. (2008). “8 - how do histological stains work?” in Theory and practice of histological techniques. eds. Bancroft J. D. Gamble M.. 6th ed (Edinburgh: Churchill Livingstone), 105–119.

[ref44] Hughes H. K. Rose D. Ashwood P. (2018). The gut microbiota and dysbiosis in autism spectrum disorders. Curr. Neurol. Neurosci. Rep. 18:81. doi: 10.1007/s11910-018-0887-6 30251184 PMC6855251

[ref45] Iglesias-Vázquez L. Van Ginkel Riba G. Arija V. Canals J. (2020). Composition of gut microbiota in children with autism Spectrum disorder: a systematic review and meta-analysis. Nutrients 12:792. doi: 10.3390/nu12030792, PMID: 32192218 PMC7146354

[ref46] Ikeda A. Aoki N. Kido M. Iwamoto S. Nishiura H. Maruoka R.. (2014). Progression of autoimmune hepatitis is mediated by IL-18-producing dendritic cells and hepatic CXCL9 expression in mice. Hepatology 60, 224–236. doi: 10.1002/hep.27087, PMID: 24700550

[ref47] Jaskowski T. D. Schroder C. Martins T. B. Mouritsen C. L. Litwin C. M. Hill H. R. (1996). Screening for antinuclear antibodies by enzyme immunoassay. Am. J. Clin. Pathol. 105, 468–473. doi: 10.1093/ajcp/105.4.468, PMID: 8604689

[ref48] Jerez A. Clemente M. J. Makishima H. Koskela H. LeBlanc F. Peng Ng K.. (2012). STAT3 mutations unify the pathogenesis of chronic lymphoproliferative disorders of NK cells and T-cell large granular lymphocyte Leukemia. Blood 120, 3048–3057. doi: 10.1182/blood-2012-06-435297, PMID: 22859607 PMC3471515

[ref49] Jiang C.-C. Lin L.-S. Long S. Ke X.-Y. Fukunaga K. Lu Y.-M.. (2022). Signalling pathways in autism Spectrum disorder: mechanisms and therapeutic implications. Sig. Transduct Target Ther. 7, 1–36. doi: 10.1038/s41392-022-01081-0 PMC9273593 35817793

[ref50] Kalinichenko L. S. Mühle C. Jia T. Anderheiden F. Datz M. Eberle A.-L.. (2021). Neutral sphingomyelinase mediates the co-morbidity Trias of alcohol abuse, major depression and bone defects. Mol. Psychiatry 26, 7403–7416. doi: 10.1038/s41380-021-01304-w, PMID: 34584229 PMC8872992

[ref51] Kamo N. Ke B. Ghaffari A. A. Shen X. Busuttil R. W. Cheng G.. (2013). ASC/Caspase-1/IL-1β Signaling triggers inflammatory responses by promoting HMGB1 induction in liver ischemia/reperfusion injury. Hepatology 58, 351–362. doi: 10.1002/hep.26320, PMID: 23408710 PMC3679353

[ref52] Kang D.-W. Adams J. B. Coleman D. M. Pollard E. L. Maldonado J. McDonough-Means S.. (2019). Long-term benefit of microbiota transfer therapy on autism symptoms and gut microbiota. Sci. Rep. 9:5821. doi: 10.1038/s41598-019-42183-0 30967657 PMC6456593

[ref53] Kang D.-W. Adams J. B. Gregory A. C. Borody T. Chittick L. Fasano A.. (2017). Microbiota transfer therapy alters gut ecosystem and improves gastrointestinal and autism symptoms: an open-label study. Microbiome 5:10. doi: 10.1186/s40168-016-0225-7 28122648 PMC5264285

[ref54] Katz-Agranov N. Zandman-Goddard G. (2017). The microbiome and systemic lupus erythematosus. Immunol. Res. 65, 432–437. doi: 10.1007/s12026-017-8906-2, PMID: 28233089

[ref55] Kawasumi H. Gono T. Kawaguchi Y. Kaneko H. Katsumata Y. Hanaoka M.. (2014). IL-6, IL-8, and IL-10 are associated with Hyperferritinemia in rapidly progressive interstitial lung disease with polymyositis/dermatomyositis. Biomed. Res. Int. 2014:815245. doi: 10.1155/2014/815245, PMID: 24800252 PMC3988788

[ref56] Koch-Edelmann S. Banhart S. Saied E. M. Rose L. Aeberhard L. Laue M.. (2017). The cellular ceramide transport protein CERT promotes *Chlamydia Psittaci* infection and controls bacterial sphingolipid uptake. Cell. Microbiol. 19:e12752. doi: 10.1111/cmi.12752, PMID: 28544656

[ref57] Kumar M. Dandapat S. Sinha M. Kumar A. Raipat B. (2017). Different blood collection methods from rats: a review. Balneo PRM Res. J. 8, 46–50. doi: 10.12680/balneo.2017.141, PMID: 37511267

[ref58] Kummen M. Hov J. R. (2019). The gut microbial influence on cholestatic liver disease. Liver Int. 39, 1186–1196. doi: 10.1111/liv.14153, PMID: 31125502

[ref59] Lang J. Price A. B. Levi A. J. Burke M. Gumpel J. M. Bjarnason I. (1988). Diaphragm disease: pathology of disease of the small intestine induced by non-steroidal anti-inflammatory drugs. J. Clin. Pathol. 41, 516–526. doi: 10.1136/jcp.41.5.516 3384981 PMC1141503

[ref60] LaRusso N. F. Tabibian J. H. O’Hara S. P. (2017). Role of the intestinal microbiome in cholestatic liver disease. Dig. Dis. 35, 166–168. doi: 10.1159/000450906, PMID: 28249266 PMC5941303

[ref61] Lázár B. László S. B. Hutka B. Tóth A. S. Mohammadzadeh A. Berekméri E.. (2021). A comprehensive time course and correlation analysis of indomethacin-induced inflammation, bile acid alterations and dysbiosis in the rat small intestine. Biochem. Pharmacol. 190:114590. doi: 10.1016/j.bcp.2021.114590, PMID: 33940029

[ref62] Lebuhn M. Derenkó J. Rademacher A. Helbig S. Munk B. Pechtl A.. (2016). DNA and RNA extraction and quantitative real-time PCR-based assays for biogas biocenoses in an interlaboratory comparison. Bioengineering (Basel) 3:7. doi: 10.3390/bioengineering3010007, PMID: 28952569 PMC5597165

[ref63] Licata A. Maida M. Cabibi D. Butera G. Macaluso F. S. Alessi N.. (2014). Clinical features and outcomes of patients with drug-induced autoimmune hepatitis: a retrospective cohort study. Dig. Liver Dis. 46, 1116–1120. doi: 10.1016/j.dld.2014.08.040, PMID: 25224696

[ref64] Linossi E. M. Calleja D. J. Nicholson S. E. (2018). Understanding SOCS protein specificity. Growth Factors 36, 104–117. doi: 10.1080/08977194.2018.1518324, PMID: 30318950

[ref65] Lintas C. Persico A. M. (2009). Autistic phenotypes and genetic testing: state-of-the-art for the clinical geneticist. J. Med. Genet. 46, 1–8. doi: 10.1136/jmg.2008.060871, PMID: 18728070 PMC2603481

[ref66] Logsdon A. F. Erickson M. A. Rhea E. M. Salameh T. S. Banks W. A. (2018). Gut reactions: how the blood-brain barrier connects the microbiome and the brain. Exp. Biol. Med. (Maywood) 243, 159–165. doi: 10.1177/1535370217743766, PMID: 29169241 PMC5788145

[ref67] Luan J. Zhang X. Wang S. Li Y. Fan J. Chen W.. (2018). NOD-like receptor protein 3 inflammasome-dependent IL-1β accelerated con A-induced hepatitis. Front. Immunol. 9:758. doi: 10.3389/fimmu.2018.00758 29692782 PMC5902503

[ref68] Lucas S. (2016). The pharmacology of indomethacin. Headache: the journal of head and face. Pain 56, 436–446. doi: 10.1111/head.12769 26865183

[ref69] Łukasik J. Patro-Gołąb B. Horvath A. Baron R. Szajewska H. (2019). Early life exposure to antibiotics and autism spectrum disorders: a systematic review. J. Autism Dev. Disord. 49, 3866–3876. doi: 10.1007/s10803-019-04093-y, PMID: 31175505 PMC6667689

[ref70] Manns M. P. Czaja A. J. Gorham J. D. Krawitt E. L. Mieli-Vergani G. Vergani D.. (2010). Diagnosis and Management of Autoimmune Hepatitis. Hepatology 51, 2193–2213. doi: 10.1002/hep.23584, PMID: 20513004

[ref71] Maseda D. Ricciotti E. (2020). NSAID–gut microbiota interactions. Front. Pharmacol. 11:1153. doi: 10.3389/fphar.2020.01153 32848762 PMC7426480

[ref72] Maseda D. Zackular J. P. Trindade B. Kirk L. Roxas J. L. Rogers L. M.. (2019). Nonsteroidal anti-inflammatory drugs Alter the microbiota and exacerbate *Clostridium Difficile* colitis while dysregulating the inflammatory response. MBio 10, e02282–e02218. doi: 10.1128/mBio.02282-18 30622186 PMC6325247

[ref73] Masi A. Breen E. J. Alvares G. A. Glozier N. Hickie I. B. Hunt A.. (2017). Cytokine levels and associations with symptom severity in male and female children with autism Spectrum disorder. Mol. Autism. 8:63. doi: 10.1186/s13229-017-0176-2 29214007 PMC5712192

[ref74] Matelski L. Van de Water J. (2016). Risk factors in autism: thinking outside the brain. J. Autoimmun. 67, 1–7. doi: 10.1016/j.jaut.2015.11.003, PMID: 26725748 PMC5467975

[ref75] Matsumoto T. Nakamura S. Esaki M. Yada S. Koga H. Yao T.. (2006). Endoscopic features of chronic nonspecific multiple ulcers of the small intestine: comparison with nonsteroidal anti-inflammatory drug-induced enteropathy. Dig. Dis. Sci. 51, 1357–1363. doi: 10.1007/s10620-006-9080-x, PMID: 16868823

[ref76] McLellan J. Kim D. H. J. Bruce M. Ramirez-Celis A. Van de Water J. (2022). Maternal immune dysregulation and autism-understanding the role of cytokines, Chemokines and Autoantibodies. Front Psychiatry. 13:834910. doi: 10.3389/fpsyt.2022.834910, PMID: 35722542 PMC9201050

[ref77] Mekori-Domachevsky E. Taler M. Shoenfeld Y. Gurevich M. Sonis P. Weisman O.. (2017). Elevated proinflammatory markers in 22q11.2 deletion syndrome are associated with psychosis and cognitive deficits. J. Clin. Psychiatry 78, e1219–e1225. doi: 10.4088/JCP.16m11207, PMID: 29141125

[ref78] Mohamed D. I. Abou-Bakr D. A. Ezzat S. F. El-Kareem H. F. A. Nahas H. H. A. Saad H. A.. (2021). Vitamin D3 prevents the deleterious effects of testicular torsion on testis by targeting MiRNA-145 and ADAM17: in silico and in vivo study. Pharmaceuticals 14:1222. doi: 10.3390/ph14121222, PMID: 34959623 PMC8703569

[ref79] Mohamed D. I. Alaa El-Din Aly El-Waseef D. Nabih E. S. El-Kharashi O. A. Abd El-Kareem H. F. Abo Nahas H. H.. (2022a). Acetylsalicylic acid suppresses alcoholism-induced cognitive impairment associated with atorvastatin intake by targeting cerebral MiRNA155 and NLRP3: in vivo, and in silico study. Pharmaceutics 14:529. doi: 10.3390/pharmaceutics14030529, PMID: 35335908 PMC8948796

[ref80] Mohamed D. I. Ezzat S. F. Elayat W. M. El-Kharashi O. A. El-Kareem H. F. A. Nahas H. H. A.. (2022b). Hepatoprotective role of carvedilol against ischemic hepatitis associated with acute heart failure via targeting MiRNA-17 and mitochondrial dynamics-related proteins: an in vivo and in silico study. Pharmaceuticals 15:832. doi: 10.3390/ph15070832 35890131 PMC9319470

[ref81] Mohamed A. S. Hosney M. Bassiony H. Hassanein S. S. Soliman A. M. Fahmy S. R.. (2020). Sodium pentobarbital dosages for exsanguination affect biochemical, molecular and histological measurements in rats. Sci. Rep. 10:378. doi: 10.1038/s41598-019-57252-7 31942001 PMC6962368

[ref82] Mostafa G. A. AL-Ayadhi L. Y. (2011). A lack of association between Hyperserotonemia and the increased frequency of serum anti-myelin basic protein auto-antibodies in autistic children. J. Neuroinflammation 8:71. doi: 10.1186/1742-2094-8-71, PMID: 21696608 PMC3142225

[ref83] Mostafa G. A. Al-Ayadhi L. Y. (2013). The possible relationship between allergic manifestations and elevated serum levels of brain specific auto-antibodies in autistic children. J. Neuroimmunol. 261, 77–81. doi: 10.1016/j.jneuroim.2013.04.003, PMID: 23726766

[ref84] Murakami M. Kamimura D. Hirano T. (2019). Pleiotropy and specificity: insights from the interleukin 6 family of cytokines. Immunity 50, 812–831. doi: 10.1016/j.immuni.2019.03.027, PMID: 30995501

[ref85] Muratori L. Lohse A. W. Lenzi M. (2023). Diagnosis and Management of Autoimmune Hepatitis. BMJ 380:e070201. doi: 10.1136/bmj-2022-070201, PMID: 36746473

[ref86] Nakajima A. Fukui T. Takahashi Y. Kishimoto M. Yamashina M. Nakayama S.. (2012). Attenuation of indomethacin-induced gastric mucosal injury by prophylactic Administration of Sake Yeast-Derived Thioredoxin. J. Gastroenterol. 47, 978–987. doi: 10.1007/s00535-012-0564-5, PMID: 22402774 PMC3443347

[ref87] O’Shea J. J. Schwartz D. M. Villarino A. V. Gadina M. McInnes I. B. Laurence A. (2015). The JAK-STAT pathway: impact on human disease and therapeutic intervention. Annu. Rev. Med. 66, 311–328. doi: 10.1146/annurev-med-051113-024537, PMID: 25587654 PMC5634336

[ref88] Ohlsson A. Walia R. Shah S. S. (2020). Ibuprofen for the treatment of patent ductus arteriosus in preterm or low birth weight (or both) infants. Cochrane Database Syst. Rev. 2:CD003481. doi: 10.1002/14651858.CD003481.pub8, PMID: 32045960 PMC7012639

[ref89] Onore C. Careaga M. Ashwood P. (2012). The role of immune dysfunction in the pathophysiology of autism. Brain Behav. Immun. 26, 383–392. doi: 10.1016/j.bbi.2011.08.007 21906670 PMC3418145

[ref90] Pan Z.-Y. Zhong H.-J. Huang D.-N. Wu L.-H. He X.-X. (2022). Beneficial effects of repeated washed microbiota transplantation in children with autism. Front. Pediatr. 10:928785. doi: 10.3389/fped.2022.928785, PMID: 35783298 PMC9249087

[ref91] Pearce S. C. Coia H. G. Karl J. P. Pantoja-Feliciano I. G. Zachos N. C. Racicot K. (2018). Intestinal in vitro and ex vivo models to study host-microbiome interactions and acute stressors. Front. Physiol. 9:1584. doi: 10.3389/fphys.2018.01584, PMID: 30483150 PMC6240795

[ref92] Pétrilli V. Dostert C. Muruve D. A. Tschopp J. (2007). The inflammasome: a danger sensing complex triggering innate immunity. Curr. Opin. Immunol. 19, 615–622. doi: 10.1016/j.coi.2007.09.002, PMID: 17977705

[ref93] Quaranta G. Sanguinetti M. Masucci L. (2019). Fecal microbiota transplantation: a potential tool for treatment of human female reproductive tract diseases. Front. Immunol. 10:2653. doi: 10.3389/fimmu.2019.02653 31827467 PMC6890827

[ref94] Rogers M. A. M. Aronoff D. M. (2016). The influence of non-steroidal anti-inflammatory drugs on the gut microbiome. Clin. Microbiol. Infect. 22, e1–178.e9. doi: 10.1016/j.cmi.2015.10.003 26482265 PMC4754147

[ref95] Ross H. E. Guo Y. Coleman K. Ousley O. Miller A. H. (2013). Association of IL-12p70 and IL-6:IL-10 ratio with autism-related Behaviors in 22q11.2 deletion syndrome: a preliminary report. Brain Behav. Immun. 31, 76–81. doi: 10.1016/j.bbi.2012.12.021, PMID: 23353117 PMC3669236

[ref96] Rossignol D. A. Frye R. E. (2021). Cerebral folate deficiency, folate receptor alpha autoantibodies and leucovorin (folinic acid) treatment in autism spectrum disorders: a systematic review and meta-analysis. J. Pers. Med. 11:1141. doi: 10.3390/jpm11111141, PMID: 34834493 PMC8622150

[ref97] Schindler C. Levy D. E. Decker T. (2007). JAK-STAT Signaling: from interferons to cytokines *. J. Biol. Chem. 282, 20059–20063. doi: 10.1074/jbc.R700016200, PMID: 17502367

[ref98] Shen K. Wei Y. Lv T. Song Y. Jiang X. Lu Z.. (2021). The expression landscape of JAK1 and its potential as a biomarker for prognosis and immune infiltrates in NSCLC. BMC Bioinformatics 22:471. doi: 10.1186/s12859-021-04379-y 34587898 PMC8482691

[ref99] Singh V. K. (2009). Phenotypic expression of autoimmune autistic disorder (AAD): a major subset of autism. Ann. Clin. Psychiatry 21, 148–161. PMID: 19758536

[ref100] Solomon D. H. Kavanaugh A. J. Schur P. H. Guidelines A. C. American College of Rheumatology Ad Hoc Committee on Immunologic Testing Guidelines (2002). Evidence-based Guidelines for the use of immunologic tests: antinuclear antibody testing. Arthritis Care Res. 47, 434–444. doi: 10.1002/art.10561 12209492

[ref101] Sotgiu S. Manca S. Gagliano A. Minutolo A. Melis M. C. Pisuttu G.. (2020). Immune regulation of neurodevelopment at the mother–foetus Interface: the case of autism. Clin. Transl. Immunol. 9:e1211. doi: 10.1002/cti2.1211, PMID: 33209302 PMC7662086

[ref102] Takeichi T. Lee J. Y. W. Okuno Y. Miyasaka Y. Murase Y. Yoshikawa T.. (2022). Autoinflammatory keratinization disease with hepatitis and autism reveals roles for JAK1 kinase hyperactivity in autoinflammation. Front. Immunol. 12:737747. doi: 10.3389/fimmu.2021.737747, PMID: 35046931 PMC8761858

[ref103] Tan C. K. Ho D. Wang L. M. Kumar R. (2022). Drug-induced autoimmune hepatitis: a minireview. World J. Gastroenterol. 28, 2654–2666. doi: 10.3748/wjg.v28.i24.2654 35979160 PMC9260871

[ref104] Wan M. W. Green J. Elsabbagh M. Johnson M. Charman T. Plummer F. (2013). The BASIS team quality of interaction between at-risk infants and caregiver at 12-15 months is associated with 3-year autism outcome: at-risk infant interaction and autism. J. Child Psychol. Psychiatr. 54, 763–771. doi: 10.1111/jcpp.12032 23227853

[ref105] Wang G. Tanaka A. Zhao H. Jia J. Ma X. Harada K.. (2021). The Asian Pacific Association for the Study of the liver clinical practice guidance: the diagnosis and Management of Patients with autoimmune hepatitis. Hepatol. Int. 15, 223–257. doi: 10.1007/s12072-021-10170-1 33942203 PMC8144150

[ref106] Wei H. Zou H. Sheikh A. M. Malik M. Dobkin C. Brown W. T.. (2011). IL-6V is increased in the cerebellum of autistic brain and alters neural cell adhesion, Migration and Synaptic Formation. J Neuroinflammation 8:52. doi: 10.1186/1742-2094-8-52, PMID: 21595886 PMC3114764

[ref107] Wree A. McGeough M. D. Inzaugarat M. E. Eguchi A. Schuster S. Johnson C. D.. (2018). NLRP3 inflammasome driven liver injury and fibrosis: roles of IL-17 and TNF in mice. Hepatology 67, 736–749. doi: 10.1002/hep.29523, PMID: 28902427 PMC5849484

[ref108] Zannetti C. Roblot G. Charrier E. Ainouze M. Tout I. Briat F.. (2016). Characterization of the inflammasome in human Kupffer cells in response to synthetic agonists and pathogens. J. Immunol. 197, 356–367. doi: 10.4049/jimmunol.1502301 27226092

[ref109] Zeman M. V. Hirschfield G. M. (2010). Autoantibodies and liver disease: uses and abuses. Can. J. Gastroenterol. 24, 225–231. doi: 10.1155/2010/431913, PMID: 20431809 PMC2864616

[ref110] Zhang Y.-L. Li Z.-J. Gou H.-Z. Song X.-J. Zhang L. (2022). The gut microbiota–bile acid Axis: a potential therapeutic target for liver fibrosis. Front. Cell. Infect. Microbiol. 12:945368. doi: 10.3389/fcimb.2022.945368, PMID: 36189347 PMC9519863

[ref111] Ziesenitz V. C. Welzel T. van Dyk M. Saur P. Gorenflo M. van den Anker J. N. (2022). Efficacy and safety of NSAIDs in infants: a comprehensive review of the literature of the past 20 years. Paediatr. Drugs 24, 603–655. doi: 10.1007/s40272-022-00514-1 36053397 PMC9592650

[ref112] Zou T. Liu J. Zhang X. Tang H. Song Y. Kong X. (2020). Autoantibody and autism Spectrum disorder: a systematic review. Res. Autism Spectr. Disord. 75:101568. doi: 10.1016/j.rasd.2020.101568, PMID: 34834493

